# Targeting the Endothelium to Achieve Cardioprotection

**DOI:** 10.3389/fphar.2021.636134

**Published:** 2021-02-02

**Authors:** Nicolas Herrera-Zelada, Ursula Zuñiga-Cuevas, Andres Ramirez-Reyes, Sergio Lavandero, Jaime A. Riquelme

**Affiliations:** ^1^ Advanced Center for Chronic Disease (ACCDiS), Facultad de Ciencias Químicas y Farmacéuticas and Facultad de Medicina, Universidad de Chile, Santiago, Chile; ^2^ Department of Internal Medicine (Cardiology Division), University of Texas Southwestern Medical Center, Dallas, TX, United States

**Keywords:** cardioprotection, endothelium, ischemia/reperfusion, endothelial cells, myocardial infarction, small extracellular vesicles

## Abstract

Despite considerable improvements in the treatment of myocardial infarction, it is still a highly prevalent disease worldwide. Novel therapeutic strategies to limit infarct size are required to protect myocardial function and thus, avoid heart failure progression. Cardioprotection is a research topic with significant achievements in the context of basic science. However, translation of the beneficial effects of protective approaches from bench to bedside has proven difficult. Therefore, there is still an unmet need to study new avenues leading to protecting the myocardium against infarction. In line with this, the endothelium is an essential component of the cardiovascular system with multiple therapeutic targets with cardioprotective potential. Endothelial cells are the most abundant non-myocyte cell type in the heart and are key players in cardiovascular physiology and pathophysiology. These cells can regulate vascular tone, angiogenesis, hemostasis, and inflammation. Accordingly, endothelial dysfunction plays a fundamental role in cardiovascular diseases, which may ultimately lead to myocardial infarction. The endothelium is of paramount importance to protect the myocardium from ischemia/reperfusion injury via conditioning strategies or cardioprotective drugs. This review will provide updated information on the most promising therapeutic agents and protective approaches targeting endothelial cells in the context of myocardial infarction.

## Introduction

Myocardial infarction (MI) is the leading cause of death worldwide (Jayaraj et al., 2018). The primary clinical treatment consists of the recovery of blood flow to the heart and a phenomenon called reperfusion. However, reperfusion itself can generate more damage, leading to cardiomyocyte death. This damage varies between patients but usually leads to some degree of heart failure (Kemp and Conte, 2012). As a result of significant improvements over the last decades, the current treatment of MI has certainly reduced mortality. Nevertheless, to attenuate the severity of post-MI heart failure, adequate protection of the myocardium is still warranted to reduce infarct size and improve ventricular function recovery after ischemia/reperfusion (I/R) injury. Several strategies have been studied to activate mechanisms within cardiac cells to minimize I/R injury. This is the core of the concept called cardioprotection, that can be understood as “*all measures and interventions to prevent, attenuate and repair myocardial injury*” (Heusch, 2020), of which different approaches have been discovered (Garrido et al., 2017; Grilo et al., 2017; Lefer and Marbán, 2017; Caricati-Neto et al., 2019). Among these, the protection of the myocardium via endothelial cells (ECs) is an attractive line of action.

The endothelium is a monolayer of cells that covers the interior of every major and minor vessel and is the first line of contact between the lumen and other tissues. It is involved in the control of vascular permeability, transport logistics, regulation of the vascular tone, mediators of the immune response, angiogenesis, hemostasis control, and endocrine and paracrine communication (Bracamonte-baran and Daniela, 2017). A healthy endothelium is important for myocardial contraction, as it improves contractility by increasing the sensitivity of myofilaments to calcium (Shen et al., 2013). Moreover, ECs are a crucial target for cardioprotection, given their paramount importance in preserving the microvasculature after I/R injury (Bell and Yellon, 2012). Also, the endothelium has been shown to exert a critical role in conditioning phenomena, given its capacity to release protective factors and express receptors for pro-survival molecules (Hernández-Reséndiza et al., 2018). Since cardioprotective therapies usually focus on the cardiomyocyte, other cell types are often left in the dark. It is essential to understand the heart as a community of different cell types. The sole focus of new therapies in one might explain the differences in the results of clinical and preclinical studies (Bell and Yellon, 2012). In this review, we provide an update on new strategies that aim for the endothelium as a cardioprotective target.

### Cardioprotection: The Long Hard Road to Translation

Before discussing novel approaches targeting ECs to limit reperfusion injury, it is important to address the current state of the cardioprotection field. The study of cardioprotective strategies in preclinical research has been very successful. Indeed, protection of the myocardium against I/R injury may be achieved by a wide variety of drugs and peptides, as well as conditioning maneuvers (Heusch, 2020). However, the translation of these findings into a clinical setting has been challenging and highly disappointing. For example, the CONDI2/ERIC-PPCI randomized trial showed that remote ischemic conditioning (RIC) was unable to reduce the infarct size in ST-elevation myocardial infarction (STEMI) patients who underwent primary percutaneous coronary intervention (PPCI) (Hausenloy et al., 2019b). The neutral results reported in this study, which evaluated more than 5,000 patients, have shaken the hopes of translating cardioprotection from animal models to clinical conditions. In this regard, Heusch (Heusch and Gersh, 2020) has provided an interesting analysis addressing this trial, indicating that besides the presence of comorbidities and comedications, there are other important variables to consider in order to understand the results of this study. The mortality of MI has been significantly reduced throughout the years as a consequence of effective reperfusion therapy, as well as the use of cardioprotective pharmacotherapy that can reduce myocardial remodeling after infarction (Heusch and Gersh, 2020). Moreover, most of the patients in this trial had no clinical signs of heart failure at randomization (more than 95% were classified as Killip class 1), cardiac mortality after 1 year was 2.7% and hospitalization for heart failure in 1 year was 7.1%. This low incidence of events may explain why additional protection from RIC was challenging to observe (Heusch and Gersh, 2020). However, the RIC-STEMI trial showed in 2018 that RIPC significantly reduced cardiac mortality and hospitalization for heart failure after follow-up, despite classifying more than 80% of patients in Killip class 1 at admission, but this trial had far fewer patients (less than 600) (Gaspar et al., 2018). Recently, the FIRST study has reported that RIPC did not reduce major adverse cardiovascular events as compared with standard care after 90 days follow-up (Cheskes et al., 2020). While this study had a follow-up of only 3 months, it evaluated 1,667 patients, which is a larger amount than RIC-STEMI (Cheskes et al., 2020). In light of these inconsistencies, there are several hypotheses attempting to explain the disconnection between bench and bedside.

### Animal Models Versus the Clinical Context

Preclinical studies provide a somewhat reductionist approach to MI, given the use of young and healthy animals. Still, these studies can reveal key mechanistic findings into how cardioprotection elicited by various compounds or strategies works. Animal models that can address comorbidities may have more translational value but less mechanistic assessment (Rossello and Yellon, 2016). Furthermore, animal models and methodologies to assess cardioprotective therapies have not been thoroughly standardized, leading to variable results and low translational value. In order to address this issue, Bøtker et al. elaborated thorough and extensive practical guidelines to achieve rigorours and reproducible results in cardioprotection studies (Bøtker et al., 2018). These guidelines suggest careful study design, randomization, blinding and selection of the right statistical anaylsis. Moreover, it also addressed infarct size assessment in preclinical and clinical settings, clear establishment of exclusion criteria for Langendorff perfused hearts, standardized protocols for isolation of adult rat and mouse ventricular cardiomyocytes, among others (Bøtker et al., 2018).

Regarding problems with translation, Heusch has previously highlighted the importance of addressing issues such as: 1) lack of clarity of how long is the window for protection by reperfusion is extended by ischemic preconditioning, or what is the maximum time of ischemia so that postconditioning can still confer cardioprotection, 2) evaluation of other end points beyond infarct size, such as assessment of myocardial remodeling and mortality, 3) problems in the design and conduction of clinical trials, 4) robustness and reproducibility of data, 4) inadequate dosing and timing of phase II trials and 5) selection of cardioprotective strategies with comprehensive preclinical data to perform clinical trials (Heusch, 2017).

Another problem that needs to be accounted for is that preclinical models only test the effects of protective treatments against MI in the short term, which impairs our ability to predict long-term effects (Heusch, 2018). On the other hand, small trials may provide a promising proof-of-concept with little mechanistic evidence. In contrast, large randomized clinical trials have great translational value but relatively little mechanistic evaluation (Rossello and Yellon, 2016). Clinical trials also have limitations. For instance, infarct size is often evaluated by released biomarkers (such as troponin T) instead of more accurate imaging methods (Heusch, 2020). Nonetheless, suitably designed trials can provide a more precise long-term assessment of clinical outcomes.

To narrow the gap between these two settings, it has been proposed that preclinical cardioprotection studies should be performed in large animals, such as pigs since they more closely resemble human cardiovascular physiology and pathophysiology. Moreover, these studies should be extended for a 1 year follow-up to determine the long-term effect of cardioprotective strategies (Heusch, 2018), but it’s important to consider that this may be extremely expensive and not routinely feasible for all laboratories. Interestingly, a recent study showed that rats treated with heparin, a platelet inhibitor and an opioid agonist -mimicking background treatment of patients-reduced the infarct size, but RIC did not confer further protection. Nevertheless, treatment with a caspase inhibitor -which acts via an independent protective pathway-elicited further infarct size reduction (He et al., 2020). This study provides an important proof-of-concept of new models to improve the translational value of preclinical studies.

### Comorbidities and Risk Factors

Obesity, sex, diabetes, aging, and smoking may impair cardioprotective therapies' effectiveness (Kleinbongard et al., 2019). MI patients may often suffer from more than one of these diseases and risk factors. Thus, it stands to reason to hypothesize this as a significant reason for the disconnection between preclinical and clinical studies. Moreover, this obstacle may be challenging to overcome, given that animal models often address one comorbidity, instead of a more elaborate setting of multiple diseases and risk factors.

### Comedications

**FIGURE 1:**
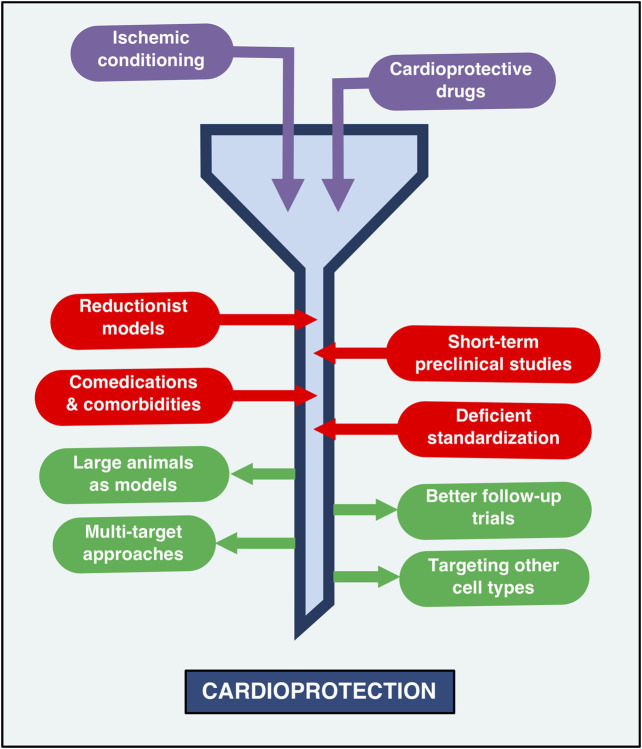
The current status of cardioprotection: Barriers and opportunities. The road to cardioprotection, while successful in preclinical settings, has been paved with obstacles and pitfalls that impede adequate clinical protection against myocardial infarction. Thus, the state of cardioprotection research can be likened to a funnel, whereby the transit of therapeutic strategies -such as remote ischemic conditioning and protective drugs-towards cardioprotection is slowed down by the use of reductionist models that lack thorough standardization, the presence of comorbidities and comedications that can impair protective therapies and preclinical models that tend to evaluate only short-term effects. The narrow part of this funnel may be enlarged by performing long-term studies in large animal models that more closely resemble the infarcted human heart. The use of multi-target approaches, as well as the targeting of other cell types (not just cardiomyocytes) has also been proposed as potentially effective strategies aimed at improving the translation of cardioprotection effectiveness in clinical contexts.

Comorbidities can impair cardioprotection, and by the same token, the administration of drugs to treat these conditions can also mask or abrogate cardioprotection generated by novel compounds or conditioning phenomena (Kleinbongard et al., 2019). Moreover, as Heusch has suggested, clinical trials often assess cardiovascular mortality after one year, as well as heart failure progression, which may be significantly influenced by the use of drugs, such as beta-blockers, angiotensin II receptor antagonists, and angiotensin-converting enzyme inhibitors, which are drugs known to be protective (Heusch, 2020). Moreover, anesthetics, such as propofol, can impair the beneficial effect of RIC, and other drugs, such as P2Y_12_ inhibitors, may actually induce cardioprotection themselves, which can mask any added protection conferred by RIPC (Kleinbongard et al., 2019). The current problems interfering with translation of cardioprotection to clinical settings and the potential strategies to enable adequate clinical protection are summarized in Figure 1. In addition to these considerations, redundant cell death mechanisms during I/R injury may warrant the use of a multi-target approach instead of only addressing one at a time (Davidson et al., 2019). This may involve not only different therapeutic targets within the cardiomyocyte but also addressing the complex interplay between cardiomyocytes and other cardiac cells. While fibroblasts are key cells in many cardioprotective strategies (Bell and Yellon, 2012), this review will focus on the targeting of the endothelium to protect the myocardium from reperfusion-induced cell death.

### The Endothelium: A Key Player in Cardioprotection

ECs are a critical component of multiple therapeutic approaches to confer protection against I/R injury. This central role is partly related to their ability to maintain vascular homeostasis. The endothelium regulates vascular tone by different mechanisms, but its mechanisms can be simplified by an equilibrium between vasodilative and vasoconstrictive stimuli. The endothelium can mediate both kinds of stimuli and produce contraction or relaxation in vascular smooth muscle cells (VSMC), therefore controlling vascular tone. On the one hand, the endothelium is capable of secreting vasodilator molecules, such as nitric oxide (NO), carbon monoxide (CO), prostacyclin (PGI_2_), bradykinin, and adenosine.

ECs can induce vasoconstriction via the release of endothelin-1, angiotensin II, thromboxane A_2,_ and reactive oxygen species (ROS) (Krüger-Genge et al., 2019). An imbalance between vasoconstriction and vasodilatation is related to endothelial dysfunction. In a dysfunctional state, there is a reduction in the bioavailability of NO, a lesser response to vasodilators in the vascular smooth muscle, more sensitivity to vasoconstrictors substances and a pro-inflammatory state (Steyers and Miller, 2014; Konukoglu and Uzun, 2016; Engin, 2017). A dysfunctional state of the endothelium may be triggered by an inflammatory stimulus. This stimulus can be both an innate immune response and an accumulation of ROS. The innate immune response triggers the production of TNF-alpha, a master pro-inflammatory cytokine that can activate the nuclear factor kappa-light-chain-enhancer of activated B cells (NFkB) pathway in ECs. This activation leads to the expression of adhesion molecules such as ICAM-1 or VCAM-1 (Madge and Pober, 2001). Moreover, it has been reported that TNF-alpha can potentiate the vasoconstrictive effect of serotonin in the coronary artery after stenting (Kleinbongard et al., 2011). Also, activation of the TNF-alpha receptor 1 (TNFR1) can inhibit the endothelial nitric oxide synthase (eNOS) (Pober, 2002). Importantly, NO also promotes an anti-thrombotic state, inhibiting platelet aggregation (Gries et al., 1998; Sylman et al., 2013), and thus, reduced NO bioavailability is associated with a pro-thrombotic state. The combined effect of these pro-inflammatory and thrombogenic states and the exacerbated ROS production ultimately leads to endothelial dysfunction, which is a key feature of atherosclerosis and the consequent MI.

Considering that blood-borne factors need to diffuse through the endothelium to reach the myocardium, ECs are a critical variable to consider in multiple protective strategies, such as those that aim at conditioning cardiac tissue (Bell and Yellon, 2012). In this context, to generate remote ischemic preconditioning-induced protection, a pro-survival signal needs to be produced and transported through the nervous and circulatory systems to the heart. Consequently, signaling pathways are activated in cardiomyocytes, conferring protection against I/R injury (Hernández-Reséndiza et al., 2018). Endothelial-derived NO is a powerful gaseous second messenger that can limit I/R damage. NO decreases myocardial oxygen consumption, which is a common pharmacologic effect of organic nitrates used to treat angina to prevent MI (Münzel et al., 2013). Besides, NO can also attenuate reperfusion-induced cardiomyocyte death by activating cyclic GMP-dependent kinase or by directly nitrosylating proteins (Cohen et al., 2010; Inserte and Garcia-Dorado, 2015).

Regarding cardiac ischemic conditioning, it has been recently observed in a prospective, randomized clinical trial that the endothelium may contribute to the protective effects of RIC (Corcoran et al., 2018). This study was carried out in 60 patients with stable coronary artery disease, and its findings show that RIC reduced vasoconstriction induced by increasing intracoronary acetylcholine doses as compared with sham group. There were no differences in endothelial function parameters between groups, suggesting that questions about the mechanisms mediating this effect remain unanswered (Corcoran et al., 2018). Nonetheless, previous studies have addressed potential pathways by which RIPC may induce vasodilation. For instance, the gap junction protein connexin 43 (Cx43) is a highly relevant connexin in cardiomyocytes, and studies suggest that ECs express this protein and contributes to endothelial-derived hyperpolarizing factor-induced vasodilation (Karagiannis et al., 2004). RIPC restores Cx43 expression and phosphorylation (Brandenburger et al., 2014), but whether Cx43 is essential for the reduction of infarct size elicited by RIPC remains to be elucidated.

Regarding the mechanisms mediating RIPC-induced cardioprotection, there is currently an understanding that humoral and neuronal pathways are involved in this complex response (Heusch, 2020). Considering multiple evidence, NO has been argued to be essential for early and late phase RIPC (Aggarwal et al., 2016), highlighting a potentially relevant role for the endothelium. In line with this, it has been shown that RIPC in mice promotes the eNOS-dependent production of NO, which is then oxidized to nitrites. Then, circulating nitrites diffuse into the myocardium, are subsequently reduced to NO and limit the infarct size after *in vivo* I/R (Rassaf et al., 2014). Also, it has been shown that the reduction of infarct size conferred by RIC and glyceryl trinitrate in isolated rat hearts subjected to I/R injury is diminished upon co-administration with NO or ROS scavengers (Hauerslev et al., 2018). Furthermore, the individual cardioprotective effect of these therapies is lost when they are co-administered. This observation was further confirmed by measuring endothelial function in humans, obtaining the same results, thereby suggesting an interaction between these two therapeutic strategies that may involve the participation of nitric oxide (Hauerslev et al., 2018).

**FIGURE 2:**
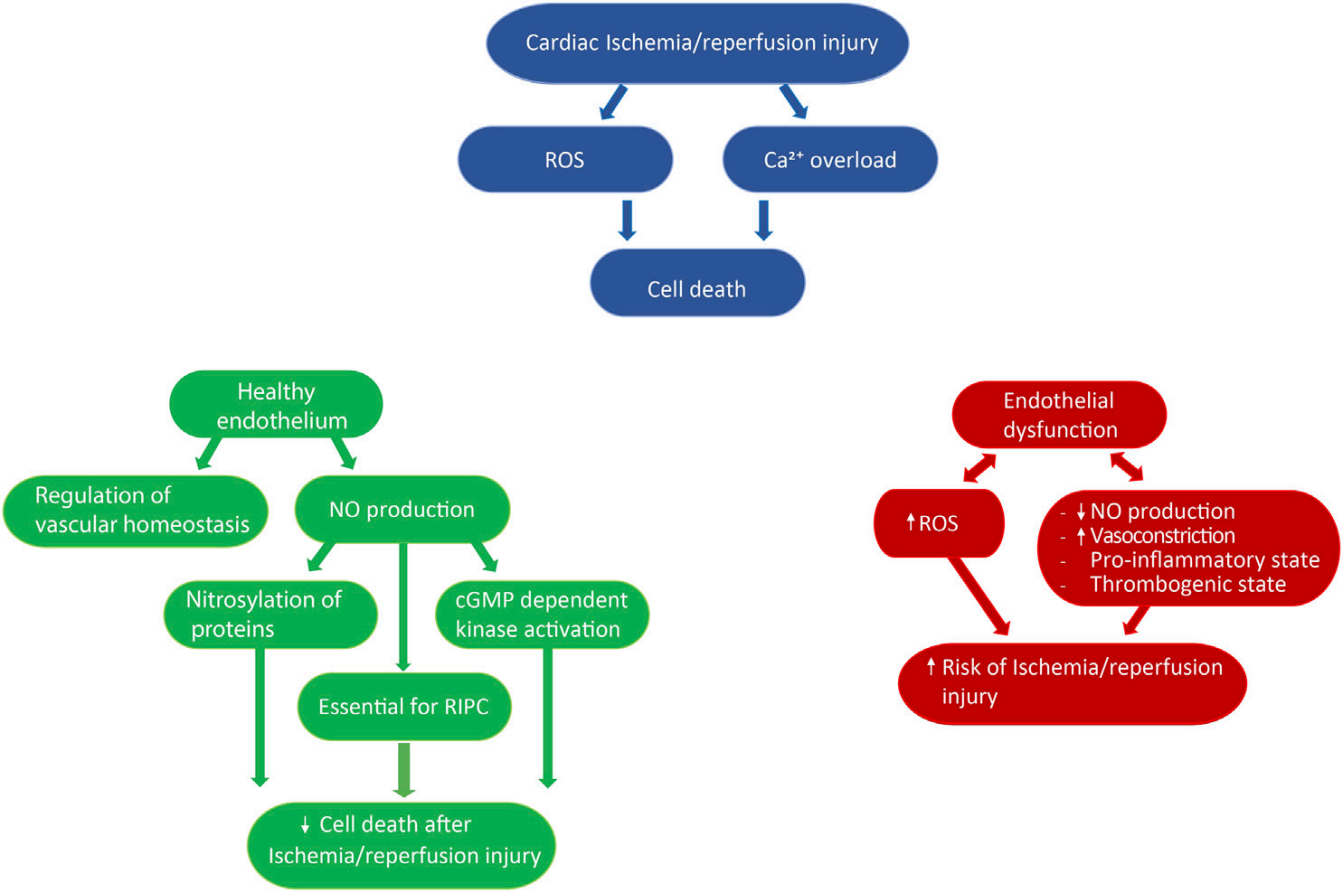
Role of the endothelium in cardioprotection. Ischemia/reperfusion injury is associated with increased reactive oxygen species production and Ca^2+^ overload, which ultimately leads to cell death (blue diagram). The endothelium has been described to both precipitate and limit ischemia/reperfusion injury, depending on the physiological state of endothelial cells and/or external stimuli. On the one hand, a healthy endothelium regulates vascular homeostasis, and the production of nitric oxide has been shown to be a key component of remote ischemic preconditioning, as well as to directly reduce ischemia/reperfusion injury by nitrosylating proteins or activating cGMP dependent kinase (green diagram). On the other hand, a dysfunctional endothelium is associated with impaired nitric oxide production, increased vasoconstriction, and reactive oxygen species production, as well as the development of a pro-inflammatory and thrombogenic state, thus favoring the onset of ischemia/reperfusion injury (red diagram). ROS: reactive oxygen species, NO: nitric oxide, RIPC: remote ischemia preconditioning.

Overall, evidence suggests conditioning maneuvers may not require the endothelium to directly protect cardiomyocytes (X. Li et al., 2004; Skyschally et al., 2018) In contrast, the endothelium plays a critical role in the protection of the coronary circulation (Hausenloy et al., 2019a; Heusch, 2016, 2019) and as discussed above, ECs interact with cardiomyocytes to provide protective signals, thereby indirectly contributing to the effectiveness of ischemic conditioning strategies. For a more comprehensive discussion addressing the participation of ECs in ischemic conditioning phenomena, we encourage the readers to consult these articles: (Bell and Yellon, 2012), (Hernández-Reséndiza et al., 2018), (Aggarwal et al., 2016). A general summary of the classical role of the endothelium in cardioprotection is presented in Figure 2.

## New Insights of Endothelial-mediated Protection Against Ischemia-Reperfusion Injury

### Endothelial Cell Subpopulations and Their Role in Cardioprotection

The endothelium is characterized by a prominent heterogenicity, showing considerable variations in structure, function, and mechanisms of ECs located in arteries, veins, and capillaries, as well as their presence in organs such as the brain, heart, and kidney (Aird, 2007, 2012). Furthermore, single-cell analysis has gained relevance in the last few years since it has proven to be a powerful tool to identify new cell populations and subpopulations. This technique enables the transcriptomic analysis of individual cells (Chaudhry et al., 2019). Recently, different sub-populations of ECs in various tissues have been described. Transcriptome heterogenicity of ECs from other non-cardiac tissues has been previously reported and reviewed (Kalucka et al., 2020). In regards to endothelial progenitor cells (EPC), sub-populations have also been identified, and their presence appears to vary in the context of coronary artery disease (Shaik et al., 2018), which may shed light on the current problems associated with the translational value of EPC therapy to treat MI.

A novel study found three different sub-populations of ECs in the aorta (Kalluri et al., 2019). These ECs had different localization, functionality, and expression of molecular markers. One group of ECs expressed genes associated with extracellular matrix production and cellular adhesion. The second group expressed genes related to lipoprotein management and angiogenesis, and the third group expressed marker associated with lymphatic endothelium (Kalluri et al., 2019). This suggests the provocative idea of targeting specific subpopulations of ECs to treat different diseases. In line with this, the endothelium is required for the revascularization of damaged tissue by I/R (Miquerol et al., 2015). Still, more recently, it has been observed that different types of ECs participate in angiogenesis, such as migratory tip cells and proliferative stalk cells (Dumas et al., 2020). From a preventive perspective, quiescent endothelial cells may be a more appropriate target, due to the fact that this cells regulates tone and vascular function and dysfunction of this cells can lead to several cardiovascular diseases such as atherosclerosis, stroke, MI and others (Kalucka et al., 2018). Interestingly, these three types of ECs have different metabolic signatures, which means that these cells can change their metabolism gene expression depending on the current physiological need (Dumas et al., 2020; Rohlenova et al., 2020). Therefore, the interventions that target the metabolism of different types of ECs may provide therapeutic advantages in pathological contexts, such as reducing myocardial damage after infarction.

Single-cell analysis in the cardiovascular system has not been limited only to physiological settings. The study by Li *et al.* performed a transcriptomic analysis of cardiac ECs from mice subjected to *in vivo* I/R injury. Their findings determined that resident ECs elicited neovascularization after infarction, with no apparent participation from endothelial-to-mesenchymal(Z. Li et al., 2019b). This study also found ten different ECs sub-populations in the mice heart, with different gene expressions between them. Additionally, the authors also report that the expression of plasmalemma vesicle-associated protein (PLVAP) was higher in ECs located in the infarct border zone in the hearts of both mice and humans (Z. Li et al., 2019b). While this study showed that Plvap is crucial for *in vitro* proliferation of ECs, *in vivo* studies are needed to confirm this observation and highlight this protein as a promising therapeutic target to boost neovascularization after MI.

Despite the encouraging results from single-cell analyses, which may open the gates to a new research field targeting specific sub-populations of ECs to protect the heart from I/R injury, these discoveries are still in infant stages. The number and type of sub-population of ECs may vary between studies, and therefore, multiple factors need to be considered, such as species, sex, age, comorbidities, among others. Thus, additional research is warranted to confirm the true potential of endothelial subpopulation-targeted therapies to exert cardioprotection.

### Cellular Therapy for Cardioprotection: Endothelial Progenitor Cells

Endothelial Progenitor Cells (EPC) contribute to vascular homeostasis and neovascularization. They are the primary endogenous vascular repair system, and their dysfunction and low levels are associated with the progression of CVD (Haider et al., 2017). Circulating EPC level rise in post-MI patients (Shintani et al., 2001), and this is associated with their vascular reparative role and, therefore, its cardioprotective role (Berezin, 2019). Stem/progenitor cell-based therapy has been extensively studied for angio-myogenic repair of the ischemic heart. Given their inherent ability to differentiate to mature ECs and release pro-angiogenic factors, EPC-based therapy is considered one of the most appropriate for vasculogenesis in the ischemic myocardium (Haider et al., 2017). Many cardioprotective treatments involving EPC have been developed. The main and most studied ones are transplantation or injection of autologous EPC, pharmacologic mobilization and potentiation of EPC, and EPC sequestration in stents (Bianconi et al., 2018). The limitations and advantages of these therapies are thoroughly discussed by Bianconi et al., 2018.

From a translational perspective, clinical trials of EPC injection have shown neutral results, having no beneficial effects in ventricular function or other clinical outcomes (Gyöngyösi et al., 2016). The potential problems accounting for the failure in the translation of cardioprotective effects have been previously discussed. Autologous EPC expansion is complex and can lead to the formation of a heterogeneous cell population that may have reduced angiogenic potential. Pharmacological EPC mobilization would solve these problems and potentially help treat inaccessible sites. Still, its therapeutic role is difficult to study as EPC could not be the only cell type producing the clinical effect observed in several clinical trials, such as the ones reviewed by Bianconi et al. (2018). In the case of EPC sequestration, this therapy is based on placing a stent on the region of cardiac injury, the stent contains immobilized antibodies that will capture circulating EPCs to promote their action there. This is an invasive therapy and is still in need of more studies. A more profound comprehension of EPC biology is required in order to improve stent design and consequently enhance their efficiency (Bianconi et al., 2018).

Recently, studies have aimed to recover the loss of EPC function observed in pathological conditions, such as coronary artery disease (Morrone et al., 2018) or diabetes (Fadini et al., 2006), where numbers of circulating EPC are lower than in healthy states. Consequently, their reparative capacity after infarction is reduced. Treatment of EPC with different molecules such as thymosin beta-4 (Poh et al., 2020) and the overexpression of Sonic hedgehog (Q. Xiao et al., 2019) recover the reparative properties of EPC after MI in the context of diabetes by inducing their mobilization (restoring their circulating levels) or promoting angiogenesis. Shexiang Baoxing pills -a traditional Chinese medicine for ischemic heart disease- causes this same effect of EPC mobilization and promotion of angiogenesis when there are no other co-morbidities, but its capacity to mobilize EPC post-MI in a diabetic context has not been studied yet (Huang et al., 2017). One of the most recent attempts to boost the cardioprotective function of EPC is the use of Danshensu, a soluble compound from a Chinese traditional medicinal herb called Dashen (Yin et al., 2017). Danshensu promotes neovascularization in post-MI rats by improving EPC survival under hypoxic conditions and accelerating their pro-angiogenic functions (Yin et al., 2017). The reduction in cell death may be partly mediated by Akt, whereas the improved angiogenic effect depends on the stromal-derived factor-1α (SDF-1α)/CXCR4 pathway (Yin et al., 2017). Additionally, extracts from the antlers of deers (Velvet antler) trigger EPC mobilization, thereby eliciting angiogenesis, as well as endothelial repair after MI in rats (Y. Li et al., 2019a). Nonetheless, while this study provided evidence hinting at a potential involvement of the Notch pathway, more research is needed to determine which are the bioactive cardioprotective molecules from Velvet antlers that generate this protective effect and the precise mechanism mediating them.

In order to simplify the evidence presented, we have chosen to use the concept of EPC, but it’s important to consider that at the moment, the definition of EPC is general, ambiguous and has been recently challenged (Medina et al., 2017). This research field evolves rapidly and current paradigms are tested continuously. An example of this is recent study that showed that unlike previously thought, intraembryonic endothelial cells are not originated from erythro-myeloid progenitors (Feng et al., 2020). Therefore, it’s necessary to have a better understanding and consensus of what specifically is an EPC and what subtypes of these are the ones being studied, especially in the contex of cellular therapy, as has been previously suggested (Medina et al., 2017). Accordingly, some studies aim to the utilization of specific subpopulations of EPC to generate cardioprotection. A recent study showed that treatment with human endothelial colony-forming cells (huECFC) limited the infarct size, reduced cardiac remodeling, and increase left ventricle functional recovery after MI in mice with severe combined immunodeficiency (Deutsch et al., 2020). Interestingly, whether huECFC proceeded from diabetic or non-diabetic patients did not impair their protective effect, suggesting a therapeutic application for diabetic patients. Still, this possibility needs to be confirmed experimentally.

To boost EPC stability and pharmacological delivery, new avenues have been explored. The magnetization of EPC with nanoparticle enables the direction of these cells using an external magnetic field to circumscribe their effects, reflected in a decrease in infarct size and improvement of left ventricular function after MI in rats (B. fang Zhang et al., 2019). Other alternatives to increase EPC retention may involve the use of hydrogels, given that administration of EPC carried by shear-thinning hyaluronic acid hydrogels was evidenced to increase retention of these cells in the myocardium as to reduce myocardial remodeling and improve functional recovery after infarction in rats (Gaffey et al., 2019). Taken together, the current evidence shows that endothelial cellular therapy has provided promising results in the preclinical arena. However, its translation from bench to bedside still needs fine-tuning before it can be a reality.

### Preconditioning by Exercise: Role of the Endothelium

Exercise is one of the primary means of protection against I/R injury and heart diseases and is related with reduction of several associated risk factors (Shephard and Balady, 1999). Throughout weeks and years, exercise can make structural and functional changes in the physiology of the heart and vasculature that can ultimately improve tolerance to cardiovascular events (Thijssen et al., 2018). This beneficial effect may occur due to the fact that exercise elicits similar cardioprotective effects to those observed in ischemic preconditioning (Penna et al., 2020). Thus, exercise is a multifactorial source of protection that activates multiple cellular pathways that can reduce cardiomyocyte death produced by I/R injury (Chowdhury et al., 2019; Penna et al., 2020). The vasculature is one of the main structures that can benefit from exercise, and the lack of exercise is correlated to a less functional endothelium that can lead to heart disease due to a rise in ROS (Durand and Gutterman, 2014). Exercise can exert a beneficial effect in endothelial function by improving skin microcirculation in patients with ischemic heart disease, highlighting its cardioprotective role as a non-pharmacological approach (Szyguła et al., 2020). Exercise improves vascular function in adolescent (Naylor et al., 2016) and adult (Qiu et al., 2018) patients with type 2 diabetes and reduce blood pressure in patients with essential hypertension by a mechanism that involves DNA methylation (Ferrari et al., 2019). Moreover, another study revealed that aged sedentary rats developed diastolic dysfunction, impaired endothelial-dependent vasodilation of coronary arterioles, and increased aortic stiffness compared to their young counterparts (Hotta et al., 2017). Still, these parameters were significantly improved after ten weeks of exercise (Hotta et al., 2017). Furthermore, the microvascular endothelial function has been shown to be impaired in obese patients. This effect is mediated by increased ROS production by NADPH oxidase in skeletal muscle and eight weeks of aerobic exercise attenuated these effects (La Favor et al., 2016). These findings suggest the provocative idea that exercise may restore the effectiveness of cardioprotective strategies in the context of aging and comorbidities by an endothelial-dependent mechanism. Thus, this possibility merits further research.

Interestingly, exercise has also been shown to be protective of the endothelium against I/R injury (Thijssen et al., 2019). A recent study was performed on 20 heart failure patients who underwent 12 weeks of either continuous or high-intensity interval training. Endothelial function was measured in the brachial artery before and after exercise-induced ischemia for 5 min, followed by 15 min reperfusion. Both types of exercise reduced endothelial I/R injury (Thijssen et al., 2019). Although this study lacks mechanistic insights, it suggests that exercise preconditioning may preserve endothelial function, potentially making the coronary endothelium susceptible to effective therapeutic targeting and thus, generating protection against I/R-induced myocardial damage.

One of the main benefits of exercise for ECs is the rise in NO by inducing shear stress in the vasculature (Rainer et al., 2000). The mechanisms involved in exercise-induced NO production by the endothelium have been previously reviewed (Adams et al., 2017). These mechanisms converge in multiple kinase cascades that stimulates eNOS activity and increase generation of NO by shear stress, due to increased blood flow as a product of exercise (Adams et al., 2017). In addition, exercise can reduce the myocardial infarct size after *in vivo* I/R injury by increasing eNOS activity and coupling, which in turn is mediated by a β3-adrenergic receptor/AMP-activated protein kinase signaling pathway (Barr et al., 2017). However, another study showed that exercise protects the myocardium from MI in obese and diabetic mice, but while this effect was associated with reduced inducible nitric oxide synthase (iNOS), it was independent of β3-adrenergic receptor (Kleindienst et al., 2016), suggesting that exercise-induced cardioprotection is a complex phenomenon and much research needs to be performed to altogether remove the veil covering pro-survival pathways in different physiological or pathophysiological contexts.

In line with this, the study by Farah *et al.* was aimed to elucidate whether eNOS-induced protection against I/R injury elicited by exercise originated from cardiomyocytes or coronary ECs, given that both cell types express this enzyme (Farah et al., 2017). Their work showed that inactivation of coronary ECs abrogated exercise-induced reduction of infarct size and left ventricle functional recovery (Farah et al., 2017).

Interestingly, the role of eNOS in exercise-mediated cardioprotection does not appear to be limited to ECs. In this context, shear stress generated by exercise also affects other cell types like red blood cells, which can also produce and carry NO (Kleinbongard et al., 2006) (Erkens et al., 2017), since these cells also have a functional eNOS (Cortese-Krott et al., 2012). Red blood cells sense shear stress and lead the entry of calcium through the Piezo-1 calcium channel, which activates eNOS in red blood cells and leads to a rise in the NO levels (Suvorava and Cortese-Krott, 2018).

NO is not the only product that can be produced by exercise, the term “exerkine” has been used since 2016 to address several peptides and nucleic acids that can be released by many tissues during exercise and wield the potential to treat metabolic (Safdar et al., 2016) or cardiovascular diseases (CVD) (Yu et al., 2017). Exerkines may be released by cardiomyocytes, fibroblasts, and ECs and include micro RNAs (miRNAs), long non-coding RNAs (lncRNAs), Brain-derived neurotrophic factor (BDNF), neuregulin (NRG), among others (Guo et al., 2020). For instance, Hou *et al.* reported that four weeks of swim exercise-induced the release of exosomal miR-342-5p, which attenuated myocardial I/R injury in rats (Hou et al., 2019). While this study does not confirm the mechanism, it does show that this exerkine may reduce I/R-induced apoptosis and enhance the activation of pro-survival kinase Akt and that exercise or laminar shear stress increases the synthesis of miR-342-5p in ECs (Hou et al., 2019). Further investigations are needed to fully identify, understand, and manipulate exerkines to harness their full power to achieve cardioprotection.

### Sex Differences in Endothelial-Mediated Cardioprotection

Males and females may express different levels of specific signaling mechanisms and thus should be thoroughly reported. Unfortunately, while this vital variable has gained more visibility in the scientific community in recent years, there is still under-reporting of sex in cellular studies. A recent meta-analysis assessed 228 studies made in cultured cells from several models including human and different animal species, published in 16 peer-reviewed cardiovascular journals, and sex was reported in 38.6% of these articles (Vallabhajosyula et al., 2020). Moreover, 54.5% of the studies used cells from only males, whereas 32.9% used male and female animals (Vallabhajosyula et al., 2020).

It has been observed since the 1980s that the incidence of CVD is markedly different between males and females. Early studies show that, on average, males are more affected by CVD than females (Bassuk and Manson, 2010). These differences are associated with sex hormones since this sex discrepancy is abrogated when post-menopausal females are compared with males of the same age (Regitz-Zagrosek and Kararigas, 2017). This suggested two important caveats to consider in cardioprotection studies focused on sex differences: age and sex hormones. There are notable differences in the aged heart and its cardioprotective response, which have been reviewed by Boengler *et al* (Boengler et al., 2009). Females animals have a known resistance to ischemic damage, even though preclinical studies were initially focused on the use of only male individuals to elude hormonal influence in the observed results (Hundscheid et al., 2018). Estrogens have been considered a concrete source of cardioprotection in post-menopausal females (Naftolin et al., 2019). Menopausal hormone therapy is used to treat symptoms elicited by the cessation of ovulation and the associated hormonal changes. It is essential to consider that the cardioprotective effects of the menopausal hormone therapy depend on the timing of administration of such treatment (Naftolin et al., 2019).

The vasculature is profoundly influenced by factors related to sex. Receptors for sexual hormones are expressed in vascular tissue, and it is usually assumed that estrogens and progesterone are cardioprotective, whereas androgens are not (Stanhewicz et al., 2018). Nonetheless, extensive research in this field has revealed in the past decades a more complex landscape than initially thought (Stanhewicz et al., 2018).

Estrogens can signal via three different receptors: Erα, ERβ, and G protein-coupled estrogen receptor (GPER). The first two are considered to act through a slow genomic response, whereas GPER exerts a rapid, non-genomic response. Studies show that the use of 17-β-estradiol induces vasodilation (Mügge et al., 1993; Stanhewicz et al., 2018). In this context, estrogens trigger an increase in the mRNA of eNOS and induce its activation (Hishikawa et al., 1995; MacRitchie et al., 1997). Accordingly, estradiol induces endothelial-dependent vasodilation and increases the sensitivity of the endothelium to other vasodilators, such as acetylcholine and prostaglandins (Miller and Mulvagh, 2007; Usselman et al., 2016). Mechanistically, it has been observed that estradiol reduces the expression and release of ET-1 in ECs, explaining at least in part, the protective effects of estrogens in the vasculature (Bilsel et al., 2000). Also, estrogens may counter-regulate the harmful effects of the renin-angiotensin system (RAS), thereby reducing blood pressure (Roesch et al., 2010; Xue et al., 2018). Evidence in humans is not as strong as in preclinical models, and there is conflicting evidence in the effects of angiotensin II (Ang II) in males and females. Toering *et al.* showed that male humans have more sensitivity to Ang II than females (Toering et al., 2015). These results contradict Bowyer *et al.*, which showed no differences in response to Ang II infusions (Ovize et al., 2001). This discrepancy can be attributed to the different doses of Ang II used in both studies. In preclinical models, it has been shown that angiotensin receptor type 2 (AT2R) is downregulated in male rats vs. female rats (Mishra et al., 2016). This is related to studies that suggest that testosterone amplifies the RAS increasing the Ang II levels (Y. F. Chen et al., 1992; Ellison et al., 1989). Extensive reviews have been written about the relationship between sex and RAS (Fischer et al., 2002; Sullivan, 2008), and there is a lack of evidence that can relate the role of the endothelium, sex differences, and the RAS in humans.

Androgens, mainly testosterone, act via androgen receptors, which are expressed in ECs (Torres-Estay et al., 2015). The administration of testosterone has been reported to improve endothelial-mediated vasodilation of rat thoracic aortas (Montaño et al., 2008). Testosterone regulates the endothelial function of the coronary circulation in hypertensive rats by a bradykinin/NO-dependent pathway (Arapa-Diaz et al., 2020). However, the effects of testosterone in the endothelium are still a matter of debate. Hypogonadal men treated with androgens have shown reduced NO bioavailability (Bernini et al., 2006). Furthermore, treatment of transgender men with testosterone was associated with endothelial dysfunction (Gulanski et al., 2020), which is a significant risk factor for MI. Therefore, further research is needed to establish the role of androgens in endothelial function, and additional mechanistic insights may help to develop therapies that can counter these potentially deleterious effects and thus avoid the impairment of other cardioprotective strategies.

Current knowledge about the cardioprotective potential of progesterone is less abundant as compared to estrogens, but there is evidence suggesting that progesterone may present relevant effects in the vasculature. For instance, progesterone increases vasodilation mediated by augmenting eNOS activity (Selles et al., 2001). However, in contrast to progesterone, the use of synthetic progesterone appears to be unable to induce NO production in ECs (Simoncini et al., 2004). Therefore, our understanding of the role of progesterone in endothelial-mediated cardioprotection is still in the early stages and requires more research, especially in I/R settings.

Recently, the group of Lieder *et al.* found that in Lewis rats, sex is not determinant in the cardioprotection achieved by ischemic preconditioning and remote ischemic preconditioning, challenging the role of sex in conditioning therapies (Lieder et al., 2019). It is important to note that other studies showed a better resistance to I/R damage in female hearts and that this resistance can be improved by preconditioning strategies (Ferdinandy et al., 2014). Taken together, the mentioned studies highlight the crucial role of sex differences in MI. Thus, thorough reporting of animal sex in all preclinical research is of utmost importance to accurately assess the real cardioprotective effects of different therapies. Moreover, the relationship between sex hormones and endothelial function is straightforward. A thorough understanding of this complex regulation may also enable or even boost other protective approaches to exert their beneficial effects on the damaged myocardium after infarction. To address this, it has been suggested that no only sex needs to be accounted for, but also comorbidities, as well as their treatments, thereby integrating preclinical, translational and clinical research (Perrino et al., 2020). A thorough analysis and recommendations to improve translational research associated to sex-specific comorbidities in cardioprotection has been reviewed by Perrino et al. (Perrino et al., 2020). Nonetheless, despite the increasing interest in sex differences in cardioprotection, there is still an important gap that needs to be addressed in terms of the influence of sex in endothelial-mediated cardioprotection and future research should focus in this particular aspect to harness the full therapeutic potential of ECs.

### Effect of Circadian Rhythm in Cardioprotection Mediated by the Endothelium

Circadian rhythms are 24 h oscillations in the behavior of organisms, in which their biological functions are coordinated with cycles of day and night. In mammals, this circadian rhythm is controlled by the circadian clock, which is divided into a master and peripheral circadian clock. The master clock is in the suprachiasmatic nucleus, whereas the peripheral clock is in almost every tissue that can respond to a specific stimulus (Du Pré et al., 2014; R.; Zhang et al., 2014). The light enters through the retina where it’s received by photosensors, converting light into information via the retinohypothalamic tract to the master clock, which in turn communicates with the peripheral clock by neurohumoral pathways (Du Pré et al., 2014; Crnko et al., 2019). This allows the body to regulate molecular pathways via transcriptional-translational loops that can reprogram cellular functions by expressing or inhibiting several genes (Wiesner et al., 2012). Circadian clocks can regulate the cardiovascular system, modifying the function of cardiomyocytes, fibroblasts, and ECs, thereby controlling blood pressure and heart rate, among other functions (Crnko et al., 2019). Besides, any disturbance in the 24 h circadian rhythms by environmental factors, such as pollution, ambient noise, tobacco, diet, physical activity, or endogenous factors such as anxiety, stress, and depression can lead to impaired vascular and cardiac function, inducing CVD such as heart failure, MI and arrhythmias (Crnko et al., 2018, 2019). Interestingly, circadian rhythms may affect the tolerance to myocardial infarction (Durgan et al., 2010). Moreover, cardioprotection may be achieved by treating intense light, and the endothelium seems to be an essential component in this therapy.

Period 2 (PER2) is a light-regulated circadian core protein (Oyama et al., 2017) that regulates endothelial function (Viswambharan et al., 2007). PER2 regulates miR-21 (Oyama et al., 2017), a microRNA that can bind to several proteins, decreasing cardiomyocyte apoptosis (J. Xiao et al., 2016) inflammation (Oyama et al., 2017), and increasing phosphofructokinase activity, leading to a higher glycolytic function that can limit the infarct size (Bartman et al., 2017).

A recent study elegantly showed that intense light generates PER2 amplitude enhancement, conferring protection against *in vivo* I/R injury in mice (Oyama et al., 2019). Furthermore, the authors showed that intense light-mediated cardioprotection is exerted by an adenosine and hypoxia-inducible factor 1⍺ (HIF1A) dependent mechanism. Also, tissue-specific studies with transgenic mice revealed that endothelial PER2 is responsible for reducing infarct size induced by intense light. This study also showed that intense light-induced increase in PER2 levels promotes transcriptional reprogramming of the endothelium and endothelial PER2 plays a crucial role in metabolism and barrier function. While the authors confirmed part of their findings by showing that intense light increases PER2-dependent metabolism, the real impact of this cardioprotective therapy in humans remains to be demonstrated. However, this work wields high translational value, and its implications are potentially groundbreaking (Oyama et al., 2019).

Overall, circadian rhythms can regulate endothelial function, and therefore, therapies such as light therapy, sleep therapy, or pharmacological chronotherapy targeting the regulation of clock genes expression may be a powerful cardioprotective strategy (Crnko et al., 2018) and thus, future randomized controlled clinical trials should confirm these promising findings.

### Mitochondrial Transplantation: A Role in Endothelial Cells?

In recent years, the transplant of organelles as a treatment for multiple diseases, including cancer (Elliott et al., 2012), Parkinson (Chang et al., 2016), and MI (Masuzawa et al., 2013; Kaza et al., 2017) has been gaining relevance. This interesting therapeutic approach was confirmed in humans by McCully *et al.*, which have developed a novel procedure to transplant autologous mitochondria to human damaged myocardium. This protective strategy has shown no auto-immune response and improved left ventricular function in pediatric patients with cardiac I/R injury (Emani et al., 2017). The protocol to isolate mitochondria is rapid, simple, and can be performed in less than 30 min (Preble et al., 2014). Mitochondria can then be directly injected in the damaged area of the myocardium or through vascular delivery via coronary arteries (McCully et al., 2017). Interestingly, mitochondria injected by vascular delivery accumulates in cardiomyocytes and blood vessels, but the exact mechanisms by which this occurs and how does mitochondria uptake takes place remains to be studied (Cowan et al., 2016; McCully et al., 2017). This therapeutic strategy opens the gates to a large number of possibilities and new questions. For instance, is it possible to transplant other organelles to other tissues and organs? Can we modify organelles in the laboratory to enhance their function and then transplant it into a patient? From an endothelial perspective, there are currently no studies showing mitochondrial transplantation. Would this therapy restore endothelial metabolism and, thereby, its function in I/R injury?

Nevertheless, mitochondrial transplantation is currently the subject of active debate, given it’s still unclear how mitochondria can survive to the initial overload of calcium in the extracellular environment, how extracellular mitochondria can supply ATP for myofilament contraction and how mitochondria enters the cardiomyocyte (Bertero et al., 2018). In a rabbit and a pig model, only a few mitochondria entered cardiomyocytes (Cowan et al., 2016; Kaza et al., 2017), and thus, it is thought that it may be unlikely that this few mitochondria make a significative effect to contribute to the total ATP-production in the heart. A recent study challenged the mitochondrial transplantation showing that mitochondrial cannot survive the ionic environment of blood or extracellular space (Bertero et al., 2020). As Bertero *et al.* suggests, perhaps its is not the mitochondria itself that provides the beneficial effect, but the content of permeabilized mitochondria may be the one that does (Bertero et al., 2020). Therefore, thorough functional and mechanistic preclinical studies are required to explore the full potential and safety of mitochondrial transplantation.

### Endothelial Small Extracellular Vesicles as a Potential Cardioprotective Therapy

Small extracellular vesicles (sEV) are released by most cell types and have emerged as critical mediators of intercellular communication and exert multiple therapeutic effects on damaged hearts (Davidson and Yellon, 2018). Cardiac cells can release sEV that may regulate different cell functions by delivering signals, such as proteins or non-coding RNAs, to other cells (Barile et al., 2017). In line with this, it has been reported that endothelial cells produce functional sEV (Riquelme et al., 2020).

The cardioprotective potential of sEV has gained increasing attention as they may be an alternative to cellular therapy that may exert the same or even more beneficial effects. In this context, sEV isolated from rat plasma may protect from I/R injury in different experimental models (Vicencio et al., 2015). Interestingly, the concentration of these plasma sEV was increased after RIC in both rats and humans (Vicencio et al., 2015), which has been recently confirmed in another independent study with patients (Frey et al., 2019). Frey et al., showed that RIC not only increases sEV, but also changes their miRNA profile, which included an increased expression of miR-21 -a cardioprotective miRNA-thus suggesting that sEV may contribute to the protective effects of RIC (Frey et al., 2019).

Regarding the endothelium as a source of protective sEV, Davidson *et al.* showed that endothelial sEV might attenuate adult rat cardiomyocyte damage after hypoxia/reoxygenation. Moreover, this study also reported that high concentrations of glucose seem to impair this effect, highlighting the potential role of comorbidities as key factors that can abolish the protective effects of endothelial sEV in I/R injury (Davidson et al., 2018). However, these observations need to be confirmed using robust myocardial infarction models.

While the protective effects of endothelial sEV are starting to be explored, the possibility that sEV derived from other cell types may also generate a beneficial impact on the endothelium has also been addressed. In line with this, sEV isolated from cells, such as EPC, mesenchymal stem cells (MSC), cardiac progenitor cells (CPC), and vascular progenitor cells (VPC), maybe cardioprotective by targeting the endothelium. An interesting study showed that EPC-derived extracellular vesicles loaded in a shear-thinning gel that triggers angiogenesis, as well as a marked recovery of hemodynamic parameters after *in vivo* I/R injury in rats. These vesicles were shown to be uptaken by ECs, thereby generating an angiogenic response, suggesting that using shear-thinning gel as a vehicle for sEV may provide better delivery and localization of these nanosized vesicles, therefore improving the efficacy and efficiency of their protective effects (C. W. Chen et al., 2018). In addition, studies have aimed to enhance the cardioprotective effects of sEV by increasing the concentrations of specific miRNAs within these vesicles to target other cells. Accordingly, sEV isolated from EPC were loaded with miR-210, which is involved in modulating the endothelial response to hypoxic conditions. Treatment with these extracellular vesicles reduced cell death, and improved angiogenesis of ECs subjected to hypoxia-reoxygenation, and these effects may be at least partly mediated by improving mitochondrial function (Ma et al., 2018). Additionally, sEV isolated from CPC promote angiogenesis in the mouse infarcted heart when loaded with miR-322 (Youn et al., 2019) by a Nox2-dependent mechanism (Ma et al., 2018). Furthermore, sEV isolated from MSC overexpressing SDF-1 reduced cell death and increased production of ECs post-MI in mice (Gong et al., 2019). Interestingly, sEV derived from human amniotic fluid mesenchymal stromal cells reduced the infarct size in acute MI in mice but could not mitigate cell death of adult rat cardiomyocytes after hypoxia/reoxygenation or high concentrations of H_2_O_2_ (Takov et al., 2020). Moreover, these vesicles did not trigger angiogenesis, but induced marked migration of EC, suggesting a partial effect in endothelial regeneration (Takov et al., 2020).

The use of endothelial sEV (or other sEV that act in the endothelium) may be a powerful cardioprotective approach. However, multiple methodological pitfalls need to be accounted for, such as the need for more pure populations of sEV, greater yields, increased stability, or directed targeting and delivery to the heart or a specific cell type in this organ.

### Pharmacological Targeting of the Endothelium

Pharmacological cardioprotection has also been described to require the endothelium to exert its beneficial effects in MI. Isoflurane can protect against cardiac ischemia/reperfusion. Interestingly, Leucker *et al.* showed that isoflurane reduced HL-1 cell death after hypoxia/reoxygenation. Still, the co-culture of these cells with human EC provided a further reduction of cell death by a NO-mediated mechanism (Leucker et al., 2011). Moreover, dexmedetomidine, an α2 adrenergic receptor agonist, is used as a sedative in the perioperative context exerts protection after ischemia/reperfusion in liver, brain, kidney, and heart. This drug can activate the reperfusion injury salvage kinase (RISK) pathway in whole isolated rat hearts (Ibacache et al., 2012). Interestingly, dexmedetomidine protects the myocardium through an eNOS-NO-PKG dependent pathway (Riquelme et al., 2016). These findings also showed that this drug was unable to reduce cell death of adult rat cardiomyocytes subjected to hypoxia-reoxygenation. Still, stimulation of human umbilical vein endothelial cells (HUVEC) with dexmedetomidine and subsequent co-culture with cardiomyocytes protected them from hypoxia/reoxygenation, highlighting a pivotal role for the endothelium in pharmacological cardioprotection (Riquelme et al., 2016). Additionally, the chemokine SDF-1α confers cardioprotection (Davidson et al., 2013), but recently, a study revealed more insights about its mechanisms. SDF-1α exerts its effects through the CXCR4 receptor, and this study showed that this chemokine was unable to reduce the infarct size in endothelial-specific CXCR4-knock out mice (Bromage et al., 2019). Moreover, the authors propose that SDF-1α may protect from I/R injury by activating the risk pathway in the endothelium, but this was only tested in HUVEC (Bromage et al., 2019). Therefore, cause-effect experiments using these transgenic mice are required to confirm this possibility.

Overall, while drug-mediated protection against MI has often been disappointing (Heusch, 2020), pharmacological targeting of the endothelium may be a new and potent coadjutant treatment to boost other cardioprotective therapies, which is in accordance to the previously suggested multi-target approach to protect the heart from I/R injury (Davidson et al., 2019). Thus, new agents (or old ones with new purposes) should be at the center of cardioprotection research.

## Conclusions and Future Perspectives

Over the last decades, significant advances have been made in the field of cardioprotection. However, the fact that many promising therapies have not been able to cross from bench to bedside has prompted the scientific community towards new horizons that may usher a new era of effective and potent cardioprotection. While many cardioprotective strategies have been focused on cardiomyocytes, the paradigm has shifted in the last few years. Other cardiac cells, such as ECs have been in the spotlight as potential therapeutic targets.

**FIGURE 3:**
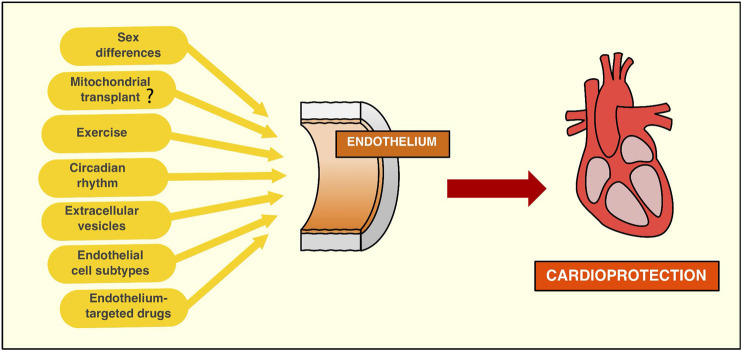
New strategies targeting the endothelium to achieve cardioprotection. **Sex differences** must be considered when it comes to research associated with endothelium targeting to protect against myocardial infarction. Evidence suggests the need to start acknowledging the endothelium as a differential component of both sexes by observing that sex hormones can influence endothelial function. While its role in cardioprotection is still underexplored, sex differences may be an important variable to take into account in order to harness the protective effects of endothelial cells. **Mitochondria transplant** to endothelial cell injury may be a promising therapy to improve the endothelial function in the context of ischemia/reperfusion context. While compelling evidence has been produced in preclinical models, the endothelium's role in this protective approach still needs to be established. **Exercise** is an effective therapy to improve the endothelial function, protecting against ischemia/reperfusion injury by nitric oxide-dependent mechanisms. More recently, the exerkines, molecules that are released during exercise, have shown beneficial effects against reperfusion-induced cardiac damage. **Circadian rhythms** can regulate endothelial function, and therefore, therapies that target the molecular machinery of the circadian rhythms can be a potent cardioprotective strategy. Recent studies show protection against cardiac ischemia/reperfusion injury by light-induced endothelial PER2 protein, highlighting the endothelium's role in circadian cardioprotection. **Endothelial small extracellular vesicles (sEV)** can limit cardiomyocytes cell death induced by ischemia/reperfusion. However, these findings need to be confirmed using *in vivo* models of myocardial infarction. Moreover, there are many methodological difficulties in the study of sEV that need to be accounted for, such as improvement in the purity of isolation methods, as well as to identify specific functions of different extracellular vesicles. **Endothelial cell subtypes** have been recently identified in the aorta by single-cell analysis. This finding suggests the interesting possibility that targeting specific subpopulations of endothelial cells may improve revascularization after myocardial ischemia/reperfusion injury, given there are subpopulations of endothelial cells more prone to angiogenesis than others. Thus, this hypothesis merits further research to explore a potential role for specific targeting of different endothelial cells. **New or old drugs** have been shown to protect the myocardium from ischemia/reperfusion injury by targeting the endothelium. While pharmacological cardioprotection has been less effective than other therapeutic approaches, it may be a powerful addition to other therapeutic agents targeting cardiomyocytes and/or fibroblasts.

The endothelium has gained significant importance in the cardioprotection field in the last few years, given its crucial role in cardiovascular physiology. Endothelial dysfunction may impact cardiomyocyte and fibroblast function. Thus, impaired endothelial integrity is linked to the development of multiple CVD, and this may partly account for the loss of cardioprotection in the presence of comorbidities. A better and more thorough understanding of the metabolism, intercellular communication, impact of sex differences, and heterogenicity of the EC populations has been achieved. Therefore, a broad range of studies has developed multiple approaches to regulate and improve endothelial function in the context of MI. Moreover, new insights in the transcriptomic and circadian regulation of ECs have shed light on new therapeutic targets, or how to adapt old ones, opening a new branch in the cardioprotection field. Thus, novel therapies, such as treatment with sEV, exercise, drugs or EPC have arisen as viable and potentially powerful cardioprotective approaches (Figure 3). The challenge today is to push this scientific evidence forward towards the clinical context. To achieve this, methodological issues and standardization of protocols still need to be addressed. For example, the safety of mitochondrial transplantation needs to be confirmed, as well as its relevance in endothelial I/R injury. The purification methods of endothelial-derived sEV need to be perfected in order to accurately attribute them the potential protective effects observed so far. A precise protocol to establish effective endothelial-dependent cardioprotection using circadian regulation in patiens is also an important future task. In addition, reproducibility in long-term large animal models must be demonstrated before many of the protective strategies addressed in this review can be translated from bench to bedside. Despite these current barriers, the endothelium is a heavyweight player in cardioprotection, and its targeting may provide potent and effective protection of the myocardium from I/R injury.

## Author Contributions

All authors participated in the conception and design of the manuscript and made significant contributions to the analysis of the evidence discussed. Besides, all authors drafted the review and gave the final approval of the submitted manuscript.

## Funding

This work was supported by grants from the Agencia Nacional de Investigacion y Desarrollo (ANID, Chile): FONDAP 15130011 (to JR and SL), FONDECYT 1200490 (SL), FONDECYT 11181000 (to JR).

## Conflict of Interest

The authors declare that this work was conducted in the absence of any commercial or financial relationships that could be construed as a potential conflict of interest.

## References

[B1] Adams V. Reich B. Uhlemann M. Niebauer J. (2017). Molecular effects of exercise training in patients with cardiovascular disease: focus on skeletal muscle, endothelium, and myocardium. Am. J. Physiol. Heart Circ. Physiol. 313 (1), H72–H88. 10.1152/ajpheart.00470.2016 28476924

[B2] Aggarwal S. Randhawa P. K. Singh N. Jaggi A. S. (2016). Preconditioning at a distance: involvement of endothelial vasoactive substances in cardioprotection against ischemia-reperfusion injury. Life Sci. 151, 250–258. 10.1016/j.lfs.2016.03.021 26979771

[B3] Aird W. C. (2007). Phenotypic heterogeneity of the endothelium: I. Structure, function, and mechanisms. Circ. Res. 100 (2), 158–173. 10.1161/01.RES.0000255691.76142.4a 17272818

[B4] Aird W. C. (2012). Endothelial cell heterogeneity. Cold Spring Harb Perspect Med. 2 (1), a006429 10.1101/cshperspect.a006429 22315715 PMC3253027

[B5] Arapa-Diaz J. C. Rouver W. D. N. Giesen J. A. S. Grando M. D. Bendhack L. M. Santos R. L. D. (2020). Testosterone increases bradykinin-induced relaxation in the coronary bed of hypertensive rats. J. Mol. Endocrinol. 65 (4), 125–134. 10.1530/JME-20-0153 33027756

[B6] Barile L. Moccetti T. Marbán E. Vassalli G. (2017). Roles of exosomes in cardioprotection. Eur. Heart J. 38 (18), 1372–1379. 10.1093/eurheartj/ehw304 27443883

[B7] Barr L. A. Lambert J. Shimizu Y. Barouch L. Naqvi N. Calvert J. (2017). Exercise training provides cardioprotection by activating and coupling endothelial nitric oxide synthase via a β3-adrenergic receptor-AMP-activated protein kinase signaling pathway. Med. Gas Res. 7 (1), 1–8. 10.4103/2045-9912.202904 28480026 PMC5402342

[B8] Bartman C. M. Oyama Y. Brodsky K. Khailova L. Walker L. Koeppen M. (2017). Intense light-elicited upregulation of miR-21 facilitates glycolysis and cardioprotection through Per2-dependent mechanisms. PLoS One 12 (4), e0176243 10.1371/journal.pone.0176243 28448534 PMC5407766

[B9] Bassuk S. S. Manson J. A. E. (2010). Gender-specific aspects of selected coronary heart disease risk factors. A summary of the epidemiologic evidence. Principles of gender-specific medicine. 2nd Edn (Amsterdam, Netherlands: Elsevier) 10.1016/B978-0-12-374271-1.00015-0

[B10] Bell R. M. Yellon D. M. (2012). Conditioning the whole heart--not just the cardiomyocyte. J. Mol. Cell. Cardiol. 53 (1), 24–32. 10.1016/j.yjmcc.2012.04.001 22521304

[B11] Berezin A. E. (2019). Endogenous vascular repair system in cardiovascular disease: the role of endothelial progenitor cells. Australas. Med. J. 12 (2). 10.21767/amj.2018.3464

[B12] Bernini G. Versari D. Moretti A. Virdis A. Ghiadoni L. Bardini M. (2006). Vascular reactivity in congenital hypogonadal men before and after testosterone replacement therapy. J. Clin. Endocrinol. Metab. 91 (5), 1691–1697. 10.1210/jc.2005-1398 16492703

[B13] Bertero E. Maack C. O’Rourke B. (2018). Mitochondrial transplantation in humans: “magical” cure or cause for concern?. J. Clin. Invest. 128 (12), 5191–5194. 10.1172/jci124944 30371508 PMC6264628

[B14] Bertero E. O'Rourke B. Maack C. (2020). Mitochondria do not survive calcium overload during transplantation. Circ. Res. 126, 784–786. 10.1161/CIRCRESAHA.119.316291 32078444 PMC7781225

[B15] Bianconi V. Sahebkar A. Kovanen P. Bagaglia F. Ricciuti B. Calabrò P. (2018). Endothelial and cardiac progenitor cells for cardiovascular repair: a controversial paradigm in cell therapy. Pharmacol. Ther., 181, 156–168. 10.1016/j.pharmthera.2017.08.004 28827151

[B16] Bilsel A. S. Moini H. Tetik E. Aksungar F. Kaynak B. Özer A. (2000). 17Beta-estradiol modulates endothelin-1 expression and release in human endothelial cells. Cardiovasc. Res. 46 (3), 579–584. 10.1016/s0008-6363(00)00046-8 10912468

[B17] Boengler K. Schulz R. Heusch G. (2009). Loss of cardioprotection with ageing. Cardiovasc. Res. 83 (2), 247–261. 10.1093/cvr/cvp033 19176601

[B18] Bøtker H. E. Hausenloy D. Andreadou I. Antonucci S. Boengler K. Davidson S. M. (2018). Practical guidelines for rigor and reproducibility in preclinical and clinical studies on cardioprotection. Basic Res. Cardiol. 113 Issue 5, 39 10.1007/s00395-018-0696-8 30120595 PMC6105267

[B19] Bracamonte-baran W. Čiháková D. (2017). Cardiac autoimmunity: myocarditis. Adv. Exp. Med. Biol. 1003, 187–221. 10.1007/978-3-319-57613-8_10 28667560 PMC5706653

[B20] Brandenburger T. Huhn R. Galas A. Pannen B. H. Keitel V. Barthel F. (2014). Remote ischemic preconditioning preserves Connexin 43 phosphorylation in the rat heart *in vivo* . J. Transl. Med. 12 (1), 228 10.1186/s12967-014-0228-8 25159820 PMC4256705

[B21] Bromage D. I. Taferner S. He Z. Ziff O. J. Yellon D. M. Davidson S. M. (2019). Stromal cell-derived factor-1α signals via the endothelium to protect the heart against ischaemia-reperfusion injury. J. Mol. Cell. Cardiol. 128, 187–197. 10.1016/j.yjmcc.2019.02.002 30738798 PMC6408335

[B22] Caricati-Neto A. Errante P. R. Menezes-Rodrigues F. S. (2019). Recent advances in pharmacological and non-pharmacological strategies of cardioprotection. Ijms 20 (16), 4002 10.3390/ijms20164002 PMC6720817 31426434

[B23] Chang J. C. Wu S. L. Liu K. H. Chen Y. H. Chuang C. S. Cheng F. C. (2016). Allogeneic/xenogeneic transplantation of peptide-labeled mitochondria in Parkinson's disease: restoration of mitochondria functions and attenuation of 6-hydroxydopamine-induced neurotoxicity. Transl. Res. 170, 40–56.e3. 10.1016/j.trsl.2015.12.003 26730494

[B24] Chaudhry F. Isherwood J. Bawa T. Patel D. Gurdziel K. Lanfear D. E. (2019). Single-cell RNA sequencing of the cardiovascular system: new looks for old diseases. Frontiers in Cardiovascular Medicine 6, 1–14. 10.3389/fcvm.2019.00173 31921894 PMC6914766

[B25] Chen Y. F. Naftilan A. J. Oparil S. (1992). Androgen-dependent angiotensinogen and renin messenger RNA expression in hypertensive rats. Hypertension 19 (5), 456–463. 10.1161/01.hyp.19.5.456 1568764

[B26] Chen C. W. Wang L. L. Zaman S. Gordon J. Arisi M. F. Venkataraman C. M. (2018). Sustained release of endothelial progenitor cell-derived extracellular vesicles from shear-thinning hydrogels improves angiogenesis and promotes function after myocardial infarction. Cardiovasc. Res. 114 (7), 1029–1040. 10.1093/cvr/cvy067 29566124 PMC5967544

[B27] Cheskes S. Koh M. Turner L. Heslegrave R. Verbeek R. Dorian P. (2020). Field implementation of remote ischemic conditioning in ST-segment-elevation myocardial infarction: the FIRST study. Can. J. Cardiol. 36 (8), 1278–1288. 10.1016/j.cjca.2019.11.029 32305146

[B28] Chowdhury M. A. Sholl H. K. Sharrett M. S. Haller S. T. Cooper C. C. Gupta R. (2019). Exercise and cardioprotection: a natural defense against lethal myocardial ischemia-reperfusion injury and potential guide to cardiovascular prophylaxis. J. Cardiovasc. Pharmacol. Therapeut. 24 (1), 18–30. 10.1177/1074248418788575 PMC7236859 30041547

[B29] Cohen M. V. Yang X. M. Liu Y. Solenkova N. V. Downey J. M. (2010). Cardioprotective PKG-independent NO signaling at reperfusion. Am. J. Physiol. Heart Circ. Physiol. 299 (6), H2028–H2036. 10.1152/ajpheart.00527.2010 20852051 PMC3006290

[B30] Corcoran D. Young R. Cialdella P. McCartney P. Bajrangee A. Hennigan B. (2018). The effects of remote ischaemic preconditioning on coronary artery function in patients with stable coronary artery disease. Int. J. Cardiol. 252, 24–30. 10.1016/j.ijcard.2017.10.082 29249435 PMC5761717

[B31] Cortese-Krott M. M. Rodriguez-Mateos A. Sansone R. Kuhnle G. G. Thasian-Sivarajah S. Krenz T. (2012). Human red blood cells at work: identification and visualization of erythrocytic eNOS activity in health and disease. Blood 120 (20), 4229–4237. 10.1182/blood-2012-07-442277 23007404

[B32] Cowan D. B. Yao R. Akurathi V. Snay E. R. Thedsanamoorthy J. K. Zurakowski D. (2016). Intracoronary delivery of mitochondria to the ischemic heart for cardioprotection. PloS One 11 (8), e0160889 10.1371/journal.pone.0160889 27500955 PMC4976938

[B33] Crnko S. Cour M. Van Laake L. W. Lecour S. (2018). Vasculature on the clock: circadian rhythm and vascular dysfunction. Vasc. Pharmacol. 108, 1–7. 10.1016/j.vph.2018.05.003 29778521

[B34] Crnko S. Du Pré B. C. Sluijter J. P. G. Van Laake L. W. (2019). Circadian rhythms and the molecular clock in cardiovascular biology and disease. Nat. Rev. Cardiol. 16 (7), 437–447. 10.1038/s41569-019-0167-4 30796369

[B35] Davidson S. M. Ferdinandy P. Andreadou I. Bøtker H. E. Heusch G. Ibáñez B. (2019). Multitarget strategies to reduce myocardial ischemia/reperfusion injury: JACC review topic of the week. J. Am. Coll. Cardiol. 73 (1), 89–99. 10.1016/j.jacc.2018.09.086 30621955

[B36] Davidson S. M. Riquelme J. A. Takov K. Vicencio J. M. Boi-Doku C. Khoo V. (2018). Cardioprotection mediated by exosomes is impaired in the setting of type II diabetes but can be rescued by the use of non-diabetic exosomes *in vitro* . J. Cell Mol. Med. 22 (1), 141–151. 10.1111/jcmm.13302 28840975 PMC5742744

[B37] Davidson S. M. Selvaraj P. He D. Boi-Doku C. Yellon R. L. Vicencio J. M. (2013). Remote ischaemic preconditioning involves signalling through the SDF-1α/CXCR4 signalling axis. Basic Res. Cardiol. 108 (5), 377 10.1007/s00395-013-0377-6 23917520

[B38] Davidson S. M. Yellon D. M. (2018). Exosomes and cardioprotection - a critical analysis. Mol. Aspect. Med. 60, 104–114. 10.1016/j.mam.2017.11.004 PMC5861305 29122678

[B39] Deutsch M.-A. Brunner S. Grabmaier U. David R. Ott I. Huber B. C. (2020). Cardioprotective potential of human endothelial-colony forming cells from diabetic and nondiabetic donors. Cells 9 (3), 588 10.3390/cells9030588 PMC7140510 32131432

[B40] Du Pré B. C. Van Veen T. A. Young M. E. Vos M. A. Doevendans P. A. Van Laake L. W. (2014). Circadian rhythms in cell maturation. Physiology 29 (1), 72–83. 10.1152/physiol.00036.2013 24382873

[B41] Dumas S. J. García-Caballero M. Carmeliet P. (2020). Metabolic signatures of distinct endothelial phenotypes. Trends Endocrinol. Metabol. 31 (8), 580–595. 10.1016/j.tem.2020.05.009 32622584

[B42] Durand M. J. Gutterman D. D. (2014). Exercise and vascular function: how much is too much?. Can. J. Physiol. Pharmacol. 92 (7), 551–557. 10.1139/cjpp-2013-0486 24873760 PMC4398063

[B43] Durgan D. J. Pulinilkunnil T. Villegas-Montoya C. Garvey M. E. Frangogiannis N. G. Michael L. H. (2010). Short communication: ischemia/reperfusion tolerance is time-of-day-dependent: mediation by the cardiomyocyte circadian clock. Circ. Res. 106 (3), 546–550. 10.1161/CIRCRESAHA.109.209346 20007913 PMC3021132

[B44] Elliott R. L. Jiang X. P. Head J. F. (2012). Mitochondria organelle transplantation: introduction of normal epithelial mitochondria into human cancer cells inhibits proliferation and increases drug sensitivity. Breast Canc. Res. Treat. 136 (2), 347–354. 10.1007/s10549-012-2283-2 23080556

[B45] Ellison K. E. Ingelfinger J. R. Pivor M. Dzau V. J. (1989). Androgen regulation of rat renal angiotensinogen messenger RNA expression. J. Clin. Invest. 83 (6), 1941–1945. 10.1172/JCI114102 2723066 PMC303916

[B46] Emani S. M. Piekarski B. L. Harrild D. del Nido P. J. McCully J. D. (2017). Autologous mitochondrial transplantation for dysfunction after ischemia-reperfusion injury. J. Thorac. Cardiovasc. Surg. 154 (1), 286–289. 10.1016/j.jtcvs.2017.02.018 28283239

[B47] Engin A. (2017). Endothelial dysfunction in obesity. Obesity and Lipotoxicity, Berlin, Germany: Springer 960, 345–379. 10.1007/978-3-319-48382-5_15 28585207

[B48] Erkens R. Suvorava T. Kramer C. M. Diederich L. D. Kelm M. Cortese-Krott M. M. (2017). Modulation of local and systemic heterocellular communication by mechanical forces: a role of endothelial nitric oxide synthase. Antioxidants Redox Signal. 26 (16), 917–935. 10.1089/ars.2016.6904 PMC5455615 27927026

[B49] Fadini G. P. Sartore S. Schiavon M. Albiero M. Baesso I. Cabrelle A. (2006). Diabetes impairs progenitor cell mobilisation after hindlimb ischaemia-reperfusion injury in rats. Diabetologia 49 (12), 3075–3084. 10.1007/s00125-006-0401-6 17072586

[B50] Farah C. Nascimento A. Bolea G. Meyer G. Gayrard S. Lacampagne A. (2017). Key role of endothelium in the eNOS-dependent cardioprotection with exercise training. J. Mol. Cell. Cardiol. 102, 26–30. 10.1016/j.yjmcc.2016.11.008 27866931

[B51] Feng T. Gao Z. Kou S. Huang X. Jiang Z. Lu Z. (2020). No evidence for erythro-myeloid progenitor-derived vascular endothelial cells in multiple organs. Circ. Res. 127 (10), 1221–1232. 10.1161/circresaha.120.317442 32791884

[B52] Ferdinandy P. Hausenloy D. J. Heusch G. Baxter G. F. Schulz R. (2014). “Interaction of risk factors, comorbidities, and comedications with ischemia/reperfusion injury and cardioprotection by preconditioning, postconditioning, and remote conditioning,” Pharmacological reviews. Editor Levy F. O., 66(4), 1142–1174. 10.1124/pr.113.008300 25261534

[B53] Ferrari L. Vicenzi M. Tarantini L. Barretta F. Sironi S. Baccarelli A. A. (2019). Effects of physical exercise on endothelial function and DNA methylation. Int. J. Environ. Res. Publ. Health 16 (14), 1–11. 10.3390/ijerph16142530 PMC6678332 31315170

[B54] Fischer M. Baessler A. Schunkert H. (2002). Renin angiotensin system and gender differences in the cardiovascular system. Cardiovasc. Res. 53 (3), 672–677. 10.1016/s0008-6363(01)00479-5 11861038

[B55] Frey U. H. Klaassen M. Ochsenfarth C. Murke F. Thielmann M. Kottenberg E. (2019). Remote ischaemic preconditioning increases serum extracellular vesicle concentrations with altered micro-RNA signature in CABG patients. Acta Anaesthesiol. Scand. 63 (4), 483–492. 10.1111/aas.13296 30548252

[B56] Gaffey A. C. Chen M. H. Trubelja A. Venkataraman C. M. Chen C. W. Chung J. J. (2019). Delivery of progenitor cells with injectable shear-thinning hydrogel maintains geometry and normalizes strain to stabilize cardiac function after ischemia. J. Thorac. Cardiovasc. Surg. 157 (4), 1479–1490. 10.1016/j.jtcvs.2018.07.117 30579534 PMC7769598

[B57] Garrido V. Mendoza-Torres E. Riquelme J. A. Díaz A. Pizarro M. Bustamante M. (2017). Novel therapies targeting cardioprotection and regeneration. Curr. Pharmaceut. Des. 23 (18), 2592–2615. 10.2174/1381612823666170112122637 28079007

[B58] Gaspar A. Lourenço A. P. Pereira M. Á. Azevedo P. Roncon-Albuquerque R. Marques J. (2018). Randomized controlled trial of remote ischaemic conditioning in ST-elevation myocardial infarction as adjuvant to primary angioplasty (RIC-STEMI). Basic Res. Cardiol. 113 (3), 14–10. 10.1007/s00395-018-0672-3 29516192

[B59] Gong X. H. Liu H. Wang S. J. Liang S. W. Wang G. G. (2019). Exosomes derived from SDF1-overexpressing mesenchymal stem cells inhibit ischemic myocardial cell apoptosis and promote cardiac endothelial microvascular regeneration in mice with myocardial infarction. J. Cell. Physiol. 234 (8), 13878–13893. 10.1002/jcp.28070 30720220 PMC13483023

[B60] Gries A. Bode C. Peter K. Herr A. Böhrer H. Motsch J. (1998). Inhaled nitric oxide inhibits human platelet aggregation, P-selectin expression, and fibrinogen binding *in vitro* and *in vivo* . Circulation 97, 1481–1487. 10.1007/978-3-322-91856-7 9576429

[B61] Grilo G. A. Shaver P. R. de Castro Brás L. E. (2017). Mechanisms of cardioprotection via modulation of the immune response. Curr. Opin. Pharmacol. 33, 6–11. 10.1016/j.coph.2017.03.002 28388508 PMC11034833

[B62] Gulanski B. I. Flannery C. A. Peter P. R. Leone C. A. Stachenfeld N. S. (2020). Compromised endothelial function in transgender men taking testosterone. Clin. Endocrinol. 92 (2), 138–144. 10.1111/cen.14132 PMC6957681 31765022

[B63] Guo Y. Chen J. Qiu H. (2020). Novel mechanisms of exercise-induced cardioprotective factors in myocardial infarction. Front. Physiol. 11, 1–12. 10.3389/fphys.2020.00199 32210839 PMC7076164

[B64] Gyöngyösi M. Wojakowski W. Navarese E. P. Moye L. (2016). Meta-analyses of human cell-based cardiac regeneration therapies: controversies in meta-analyses results on cardiac cell-based regenerative studies. Circ. Res. 118 (8), 1254–1263. 10.1161/CIRCRESAHA.115.307347 27081108 PMC4834852

[B65] Haider K. H. Aziz S. Al-Reshidi M. A. (2017). Endothelial progenitor cells for cellular angiogenesis and repair: lessons learned from experimental animal models. Regen. Med. 12 (8), 969–982. 10.2217/rme-2017-0074 29215316

[B66] Hauerslev M. Mørk S. R. Pryds K. Contractor H. Hansen J. Jespersen N. R. (2018). Influence of long-term treatment with glyceryl trinitrate on remote ischemic conditioning. Am. J. Physiol. Heart Circ. Physiol. 315 (1), H150–H158. 10.1152/ajpheart.00114.2018 29569958

[B67] Hausenloy D. J. Chilian W. Crea F. Davidson S. M. Ferdinandy P. Garcia-Dorado D. (2019a). The coronary circulation in acute myocardial ischaemia/reperfusion injury: a target for cardioprotection. Cardiovasc. Res. 115(7), 1143–1155. 10.1093/cvr/cvy286 30428011 PMC6529918

[B68] Hausenloy D. J. Kharbanda R. K. Møller U. K. Ramlall M. Aarøe J. Butler R. (2019b). Effect of remote ischaemic conditioning on clinical outcomes in patients with acute myocardial infarction (CONDI-2/ERIC-PPCI): a single-blind randomised controlled trial. Lancet 6736 (19), 1–10. 10.1016/s0140-6736(19)32039-2 PMC6891239 31500849

[B69] He Z. Davidson S. M. Yellon D. M. (2020). The importance of clinically relevant background therapy in cardioprotective studies. Basic Res. Cardiol. 115, 69 10.1007/s00395-020-00830-y 33188438 PMC7666584

[B70] Hernández-Reséndiza S. Muñoz-Vegaf M. Contreras W. E. Crespo-Avilan G. E. Rodriguez-Montesinose J. Arias-Carrión O. (2018). Responses of endothelial cells towards ischemic conditioning following acute myocardial infarction. Cond Med 1 (5), 247–258 30338315 PMC6191189

[B71] Heusch G. (2016). The coronary circulation as a target of cardioprotection. Circ. Res. 118 (10), 1643–1658. 10.1161/circresaha.116.308640 27174955

[B72] Heusch G. (2017). Critical issues for the translation of cardioprotection. Circ. Res. 120 (9), 1477–1486. 10.1161/circresaha.117.310820 28450365

[B73] Heusch G. (2018). Cardioprotection research must leave its comfort zone. Eur. Heart J. 39 (36), 3393–3395. 10.1093/eurheartj/ehy253 29722801

[B74] Heusch G. (2019). Coronary microvascular obstruction: the new Frontier in cardioprotection. Basic Res. Cardiol. 114 Issue 6, 45 10.1007/s00395-019-0756-8 31617010

[B75] Heusch G. Gersh B. J. (2020). Is cardioprotection salvageable?. Circulation 141 (6), 415–417. 10.1161/circulationaha.119.044176 32078426

[B76] Heusch G. (2020). Myocardial ischaemia-reperfusion injury and cardioprotection in perspective. Nat. Rev. Cardiol. 17 (12), 773–789. 10.1038/s41569-020-0403-y 32620851

[B77] Hishikawa K. Nakaki T. Marumo T. Suzuki H. Kato R. Saruta T. (1995). Up-regulation of nitric oxide synthase by estradiol in human aortic endothelial cells. FEBS Lett. 360 (3), 291–293. 10.1016/0014-5793(95)00124-r 7533729

[B78] Hotta K. Chen B. Behnke B. J. Ghosh P. Stabley J. N. Bramy J. A. (2017). Exercise training reverses age-induced diastolic dysfunction and restores coronary microvascular function. J. Physiol. (Lond.) 595 (12), 3703–3719. 10.1113/JP274172 28295341 PMC5471361

[B79] Hou Z. Qin X. Hu Y. Zhang X. Li G. Wu J. (2019). Longterm exercise-derived exosomal miR-342-5p: a novel exerkine for cardioprotection. Circ. Res. 124 (9), 1386–1400. 10.1161/circresaha.118.314635 30879399

[B80] Huang F. Liu Y. Yang X. Che D. Qiu K. Hammock B. D. (2017). Shexiang Baoxin pills promotes angiogenesis in myocardial infarction rats via up-regulation of 20-HETE-mediated endothelial progenitor cells mobilization. Atherosclerosis 263, 184–191. 10.1016/j.atherosclerosis.2017.06.012 28646793 PMC5828780

[B81] Hundscheid I. H. R. Schellekens D. H. S. M. Grootjans J. Derikx J. P. M. Buurman W. A. Dejong C. H. C. (2018). Females are more resistant to ischemia-reperfusion-induced intestinal injury than males. Ann. Surg. 272, 1070–1079. 10.1097/sla.0000000000003167 30614877

[B82] Ibacache M. Sanchez G. Pedrozo Z. Galvez F. Humeres C. Echevarria G. (2012). Dexmedetomidine preconditioning activates pro-survival kinases and attenuates regional ischemia/reperfusion injury in rat heart. Biochim. Biophys. Acta 1822 (4), 537–545. 10.1016/j.bbadis.2011.12.013 22230708

[B83] Inserte J. Garcia-Dorado D. (2015). The cGMP/PKG pathway as a common mediator of cardioprotection: translatability and mechanism. Br. J. Pharmacol. 172 (8), 1996–2009. 10.1111/bph.12959 25297462 PMC4386977

[B84] Jayaraj J. C. Davatyan K. Subramanian S. S. Priya J. (2018). Epidemiology of myocardial infarction. Med. Clin. 91 (4), 537–552. 10.5772/intechopen.74768 PMC2537993 17640535

[B85] Kalluri A. S. Vellarikkal S. K. Edelman E. R. Nguyen L. Subramanian A. Ellinor P. T. (2019). Single-cell analysis of the normal mouse aorta reveals functionally distinct endothelial cell populations. Circulation 140 (2), 147–163. 10.1161/circulationaha.118.038362 31146585 PMC6693656

[B86] Kalucka J. Bierhansl L. Conchinha N. V. Missiaen R. Elia I. Brüning U. (2018). Quiescent endothelial cells upregulate fatty acid β-oxidation for vasculoprotection via redox homeostasis. Cell Metabol. 28 (6), 881–e13. 10.1016/j.cmet.2018.07.016 30146488

[B87] Karagiannis J. Rand M. Li C. G. (2004). Role of gap junctions in endothelium-derived hyperpolarizing factor-mediated vasodilatation in rat renal artery. Acta Pharmacol. Sin. 25 (8), 1031–1037. 15301736

[B88] Kaza A. K. Wamala I. Friehs I. Kuebler J. D. Rathod R. H. Berra I. (2017). Myocardial rescue with autologous mitochondrial transplantation in a porcine model of ischemia/reperfusion. J. Thorac. Cardiovasc. Surg. 153 (4), 934–943. 10.1016/j.jtcvs.2016.10.077 27938904

[B89] Kemp C. D. Conte J. V. (2012). The pathophysiology of heart failure. Cardiovasc. Pathol. 21 (5), 365–371. 10.1016/j.carpath.2011.11.007 22227365

[B90] Kleinbongard P. Schulz R. Rassaf T. Lauer T. Dejam A. Jax T. (2006). Red blood cells express a functional endothelial nitric oxide synthase. Blood 107 (7), 2943–2951. 10.1182/blood-2005-10-3992 16368881

[B91] Kleinbongard P. Böse D. Baars T. Möhlenkamp S. Konorza T. Schöner S. (2011). Vasoconstrictor potential of coronary aspirate from patients undergoing stenting of saphenous vein aortocoronary bypass grafts and its pharmacological attenuation. Circ. Res. 108 (3), 344–352. 10.1161/circresaha.110.235713 21183739

[B92] Kleinbongard P. Bøtker H. E. Ovize M. Hausenloy D. J. Heusch G. (2019). Co-morbidities and co-medications as confounders of cardioprotection—does it matter in the clinical setting?. Br. J. Pharmacol. 177 (23), 5252–5269. 10.1111/bph.14839 PMC7680006 31430831

[B93] Kleindienst A. Battault S. Belaidi E. Tanguy S. Rosselin M. Boulghobra D. (2016). Exercise does not activate the β3 adrenergic receptor-eNOS pathway, but reduces inducible NOS expression to protect the heart of obese diabetic mice. Basic Res. Cardiol. 111 (4), 40 10.1007/s00395-016-0559-0 27164904

[B94] Konukoglu Dildar. Uzun Hafize. (2016). Endothelial dysfunction and hypertension. Adv exp med biol. 956, 511–540. 10.1007/5584_2016_90 28035582

[B95] Krüger-Genge A. Blocki A. Franke R. P. Jung F. (2019). Vascular endothelial cell biology: an update. Int. J. Mol. Sci. 20 (18), 1–21. 10.3390/ijms20184411 PMC6769656 31500313

[B96] La Favor J. D. Dubis G. S. Yan H. White J. D. Nelson M. A. Anderson E. J. (2016). Microvascular endothelial dysfunction in sedentary, obese humans is mediated by NADPH oxidase: influence of exercise training. Arterioscler. Thromb. Vasc. Biol. 36 (12), 2412–2420. 10.1161/ATVBAHA.116.308339 27765769 PMC5123754

[B97] Lefer D. J. Marbán E. (2017). Is cardioprotection dead?. Circulation 136 (1), 98–109. 10.1161/circulationaha.116.027039 28674094 PMC5814253

[B98] Leucker T. M. Bienengraeber M. Muravyeva M. Baotic I. Weihrauch D. Brzezinska A. K. (2011). Endothelial-cardiomyocyte crosstalk enhances pharmacological cardioprotection. J. Mol. Cell. Cardiol. 51 (5), 803–811. 10.1016/j.yjmcc.2011.06.026 21791217 PMC3342532

[B99] Li X. Heinzel F. R. Boengler K. Schulz R. Heusch G. (2004). Role of connexin 43 in ischemic preconditioning does not involve intercellular communication through gap junctions. J. Mol. Cell. Cardiol. 36 (1), 161–163. 10.1016/j.yjmcc.2003.10.019 14734058

[B100] Li Y. Wang Z. Mao M. Zhao M. Xiao X. Sun W. (2019a). Velvet antler mobilizes endothelial progenitor cells to promote angiogenesis and repair vascular endothelial injury in rats following myocardial infarction. Front. Physiol. 10, 1–12. 10.3389/fphys.2018.01940 30705637 PMC6344410

[B101] Li Z. Solomonidis E. G. Meloni M. Taylor R. S. Duffin R. Dobie R. (2019b). Single-cell transcriptome analyses reveal novel targets modulating cardiac neovascularization by resident endothelial cells following myocardial infarction. Eur. Heart J. 40 (30), 2507–2520. 10.1093/eurheartj/ehz305 31162546 PMC6685329

[B102] Lieder H. R. Irmert A. Kamler M. Heusch G. Kleinbongard P. (2019). Sex is no determinant of cardioprotection by ischemic preconditioning in rats, but ischemic/reperfused tissue mass is for remote ischemic preconditioning. Phys. Rep. 7 (12), e14146 10.14814/phy2.14146 PMC6579942 31210033

[B103] Ma X. Wang J. Li J. Ma C. Chen S. Lei W. (2018). Loading MiR-210 in endothelial progenitor cells derived exosomes boosts their beneficial effects on hypoxia/reoxygeneation-injured human endothelial cells via protecting mitochondrial function. Cell. Physiol. Biochem. 46 (2), 664–675. 10.1159/000488635 29621777

[B104] MacRitchie A. N. Jun S. S. Chen Z. German Z. Yuhanna I. S. Sherman T. S. (1997). Estrogen upregulates endothelial nitric oxide synthase gene expression in fetal pulmonary artery endothelium. Circ. Res. 81 (3), 355–362. 10.1161/01.res.81.3.355 9285637

[B105] Madge L. A. Pober J. S. (2001). TNF signaling in vascular endothelial cells. Exp. Mol. Pathol. 70 (3), 317–325. 10.1006/exmp.2001.2368 11418010

[B106] Masuzawa A. Black K. M. Pacak C. A. Ericsson M. Barnett R. J. Drumm C. (2013). Transplantation of autologously derived mitochondria protects the heart from ischemia-reperfusion injury. Am. J. Physiol. Heart Circ. Physiol. 304 (7), H966–H982. 10.1152/ajpheart.00883.2012 23355340 PMC3625892

[B107] McCully J. D. Cowan D. B. Emani S. M. del Nido P. J. (2017). Mitochondrial transplantation: from animal models to clinical use in humans. Mitochondrion 34, 127–134. 10.1016/j.mito.2017.03.004 28342934

[B108] Medina R. J. Barber C. L. Sabatier F. Dignat-George F. Melero-Martin J. M. Khosrotehrani K. (2017). Endothelial progenitors: a consensus statement on nomenclature. Stem Cells Transl Med. 6 (5), 1316–1320. 10.1002/sctm.16-0360 28296182 PMC5442722

[B109] Miller V. M. Mulvagh S. L. (2007). Sex steroids and endothelial function: translating basic science to clinical practice. Trends Pharmacol. Sci. 28 (6), 263–270. 10.1016/j.tips.2007.04.004 17466385

[B110] Miquerol L. Thireau J. Bideaux P. Sturny R. Richard S. Kelly R. G. (2015). Endothelial plasticity drives arterial remodeling within the endocardium after myocardial infarction. Circ. Res. 116 (11), 1765–1771. 10.1161/circresaha.116.306476 25834185

[B111] Mishra J. S. Hankins G. D. Kumar S. (2016). Testosterone downregulates angiotensin II type-2 receptor via androgen receptor-mediated ERK1/2 MAP kinase pathway in rat aorta. J. Renin-Angiotensin-Aldosterone Syst. JRAAS 17 (4), 1–9. 10.1177/1470320316674875 PMC5465964 27765882

[B112] Montaño L. M. Calixto E. Figueroa A. Flores-Soto E. Carbajal V. Perusquía M. (2008). Relaxation of androgens on rat thoracic aorta: testosterone concentration dependent agonist/antagonist L-type Ca2+ channel activity, and 5beta-dihydrotestosterone restricted to L-type Ca2+ channel blockade. Endocrinology 149 (5), 2517–2526. 10.1210/en.2007-1288 18276759

[B113] Morrone D. Felice F. Scatena C. De Martino A. Picoi M. L. E. Mancini N. (2018). Role of circulating endothelial progenitor cells in the reparative mechanisms of stable ischemic myocardium. Int. J. Cardiol. 257, 243–246. 10.1016/j.ijcard.2017.05.070 28918896

[B114] Mügge A. Riedel M. Barton M. Kuhn M. Lichtlen P. R. (1993). Endothelium independent relaxation of human coronary arteries by 17 beta-oestradiol *in vitro* . Cardiovasc. Res. 27 (11), 1939–1942. 10.1093/cvr/27.11.1939 8287400

[B115] Münzel T. Daiber A. Gori T. (2013). More answers to the still unresolved question of nitrate tolerance. Eur. Heart J. 34 (34), 2666–2673. 10.1093/eurheartj/eht249 23864131

[B116] Naftolin F. Friedenthal J. Nachtigall R. Nachtigall L. (2019). Cardiovascular health and the menopausal woman: the role of estrogen and when to begin and end hormone treatment. F1000Res 8, 1576 10.12688/f1000research.15548.1 PMC6733383 31543950

[B117] Naylor L. H. Davis E. A. Kalic R. J. Paramalingam N. Abraham M. B. Jones T. W. (2016). Exercise training improves vascular function in adolescents with type 2 diabetes. Phys. Rep. 4 (4), 1–12. 10.14814/phy2.12713 PMC4759041 26887327

[B118] Ovize L. Brown M. A. Jones M. (2001). Vascular reactivity in men and women of reproductive age. Am. J. Obstet. Gynecol. 185 (1), 88–96. 10.1067/mob.2001.114502 11483910

[B119] Oyama Y. Bartman C. M. Gile J. Eckle T. (2017). Circadian MicroRNAs in cardioprotection. Curr. Pharmaceut. Des. 23 (25), 3723–3730. 10.2174/1381612823666170707165319 PMC5638700 28699517

[B120] Oyama Y. Bartman C. M. Bonney S. Lee J. S. Walker L. A. Han J. (2019). Intense light-mediated circadian cardioprotection via transcriptional reprogramming of the endothelium. Cell Rep. 28 (6), 1471–e11. 10.1016/j.celrep.2019.07.020 31390562 PMC6708043

[B121] Penna C. Alloatti G. Crisafulli A. (2020). Mechanisms involved in cardioprotection induced by physical exercise. Antioxidants Redox Signal. 32 (15), 1115–1134. 10.1089/ars.2019.8009 31892282

[B122] Perrino C. Ferdinandy P. Bøtker H. E. Brundel B. J. J. M. Collins P. Davidson S. M. (2020). Improving translational research in sex-specific effects of comorbidities and risk factors in ischaemic heart disease and cardioprotection: position paper and recommendations of the ESC Working Group on Cellular Biology of the Heart. Cardiovasc. Res. cvaa155 10.1093/cvr/cvaa155 PMC7820844 32484892

[B123] Pober J. S. (2002). Endothelial activation: intracellular signaling pathways. Arthritis Res. 4 Suppl 3, S109–S116. 10.1186/ar576 12110129 PMC3240152

[B124] Poh K. K. Lee P. S. S. Djohan A. H. Galupo M. J. Songco G. G. Yeo T. C. (2020). Transplantation of endothelial progenitor cells in obese diabetic rats following myocardial infarction: role of thymosin beta-4. Cells 9 (4), 1–12. 10.3390/cells9040949 PMC7226991 32290541

[B125] Preble J. M. Pacak C. A. Kondo H. MacKay A. A. Cowan D. B. McCully J. D. (2014). Rapid isolation and purification of mitochondria for transplantation by tissue dissociation and differential filtration. JoVE 91, e51682–13. 10.3791/51682 PMC4828055 25225817

[B126] Qiu S. Cai X. Yin H. Sun Z. Zügel M. Steinacker J. M. (2018). Exercise training and endothelial function in patients with type 2 diabetes: a meta-analysis. Cardiovasc. Diabetol. 17 (1), 64–12. 10.1186/s12933-018-0711-2 29720185 PMC5930739

[B127] Rainer H. Wolf A. Gielen S. (2000). Effect of exercise on coronary endothelial function in patients with coronary artery disease. N. Engl. J. Med. 1 (7), 454–460. 10.1056/NEJM200002173420702 10675425

[B128] Rassaf T. Totzeck M. Hendgen-Cotta U. B. Shiva S. Heusch G. Kelm M. (2014). Circulating nitrite contributes to cardioprotection by remote ischemic preconditioning. Circ. Res. 114 (10), 1601–1610. 10.1161/circresaha.114.303822 24643960

[B129] Regitz-Zagrosek V. Kararigas G. (2017). Mechanistic pathways of sex differences in cardiovascular disease. Physiol. Rev. 97 (1), 1–37. 10.1152/physrev.00021.2015 27807199

[B130] Riquelme J. A. Takov K. Santiago-Fernández C. Rossello X. Lavandero S. Yellon D. M. (2020). Increased production of functional small extracellular vesicles in senescent endothelial cells. J. Cell Mol. Med. 24 (8), 4871–4876. 10.1111/jcmm.15047 32101370 PMC7176858

[B131] Riquelme J. A. Westermeier F. Hall A. R. Vicencio J. M. Pedrozo Z. Ibacache M. (2016). Dexmedetomidine protects the heart against ischemia-reperfusion injury by an endothelial eNOS/NO dependent mechanism. Pharmacol. Res. 103, 318–327. 10.1016/j.phrs.2015.11.004 26607864

[B132] Roesch D. M. Tian Y. Zheng W. Shi M. Verbalis J. G. Sandberg K. (2000). Estradiol attenuates angiotensin-induced aldosterone secretion in ovariectomized rats. Endocrinology 141 (12), 4629–4636. 10.1210/endo.141.12.7822 11108277

[B133] Rohlenova K. Goveia J. García-Caballero M. Subramanian A. Kalucka J. Treps L. (2020). Single-cell RNA sequencing maps endothelial metabolic plasticity in pathological angiogenesis. Cell Metabol. 31 (4), 862–e14. 10.1016/j.cmet.2020.03.009 32268117

[B134] Rossello X. Yellon D. M. (2016). Cardioprotection: the disconnect between bench and bedside. Circulation 134 (8), 574–575. 10.1161/CIRCULATIONAHA.116.022829 27550967

[B135] Safdar A. Saleem A. Tarnopolsky M. A. (2016). The potential of endurance exercise-derived exosomes to treat metabolic diseases. Nat. Rev. Endocrinol. 12 (9), 504–517. 10.1038/nrendo.2016.76 27230949

[B136] Selles J. Polini N. Alvarez C. Massheimer V. (2001). Progesterone and 17 beta-estradiol acutely stimulate nitric oxide synthase activity in rat aorta and inhibit platelet aggregation. Life Sci. 69 (7), 815–827. 10.1016/s0024-3205(01)01174-2 11487093

[B137] Shaik M. Shaik M. Gangapatnam S. (2018). Analysis of endothelial progenitor subpopulation cells, oxidative DNA damage, and their role in coronary artery disease. Biomed Biotechnol Res J 2 (2), 136 10.4103/bbrj.bbrj_41_18

[B138] Shen X. Tan Z. Zhong X. Tian Y. Wang X. Yu B. (2013). Endocardial endothelium is a key determinant of force-frequency relationship in rat ventricular myocardium. J. Appl. Physiol. 115 (3), 383–393. 10.1152/japplphysiol.01415.2012 23703113 PMC3743009

[B139] Shephard R. J. Balady G. J. (1999). Exercise as cardiovascular therapy. Circulation 99 (7), 963–972. 10.1161/01.cir.99.7.963 10027821

[B140] Shintani S. Murohara T. Ikeda H. Ueno T. Honma T. Katoh A. (2001). Mobilization of endothelial progenitor cells in patients with acute myocardial infarction. Circulation 103 (23), 2776–2779. 10.1161/hc2301.092122 11401930

[B141] Simoncini T. Mannella P. Fornari L. Caruso A. Willis M. Y. Garibaldi S. (2004). Differential signal transduction of progesterone and medroxyprogesterone acetate in human endothelial cells. Endocrinology 145 (12), 5745–5756. 10.1210/en.2004-0510 15358673

[B142] Skyschally A. Kleinbongard P. Lieder H. Gedik N. Stoian L. Amanakis G. (2018). Humoral transfer and intramyocardial signal transduction of protection by remote ischemic perconditioning in pigs, rats, and mice. Am. J. Physiol. Heart Circ. Physiol. 315 (1), H159–H172. 10.1152/ajpheart.00152.2018 29569956

[B143] Stanhewicz A. E. Wenner M. M. Stachenfeld N. S. (2018). Sex differences in endothelial function important to vascular health and overall cardiovascular disease risk across the lifespan. Am. J. Physiol. Heart Circ. Physiol. 315 (6), H1569–H1588. 10.1152/ajpheart.00396.2018 30216121 PMC6734083

[B144] Steyers C. M. Miller F. J. (2014). Endothelial dysfunction in chronic inflammatory diseases. Int. J. Mol. Sci. 15 (7), 11324–11349. 10.3390/ijms150711324 24968272 PMC4139785

[B145] Sullivan J. C. (2008). Sex and the renin-angiotensin system: inequality between the sexes in response to RAS stimulation and inhibition. Am. J. Physiol. Regul. Integr. Comp. Physiol. 294 (4), R1220–R1226. 10.1152/ajpregu.00864.2007 18287217

[B146] Suvorava T. Cortese-Krott M. M. (2018). Exercise-induced cardioprotection via eNOS: a putative role of red blood cell signaling. Curr. Med. Chem. 25 (34), 4457–4474. 10.2174/0929867325666180307112557 29521199

[B147] Sylman J. L. Lantvit S. M. Vedepo M. C. Reynolds M. M. Neeves K. B. (2013). Transport limitations of nitric oxide inhibition of platelet aggregation under flow. Ann. Biomed. Eng. 41 (10), 2193–2205. 10.1007/s10439-013-0803-9 23563992

[B148] Szyguła R. Wierzbicka M. Sondel G. (2020). Influence of 8-week aerobic training on the skin microcirculation in patients with ischaemic heart disease. Journal of Aging Research 2020, 6–8. 10.1155/2020/4602067 PMC7199599 32399295

[B149] Takov K. He Z. Johnston H. E. Timms J. F. Guillot P. V. Yellon D. M. (2020). Small extracellular vesicles secreted from human amniotic fluid mesenchymal stromal cells possess cardioprotective and promigratory potential. Basic Res. Cardiol. 115 (3), 26–22. 10.1007/s00395-020-0785-3 32146560 PMC7060967

[B150] Thijssen D. H. J. Redington A. George K. P. Hopman M. T. E. Jones H. (2018). Association of exercise preconditioning with immediate cardioprotection: a review. JAMA Cardiol 3 (2), 169–176. 10.1001/jamacardio.2017.4495 29188285

[B151] Thijssen D. H. J. Benda N. M. M. Kerstens T. P. Seeger J. P. H. Van Dijk A. P. J. Hopman M. T. E. (2019). 12-Week exercise training, independent of the type exercise, attenuates endothelial ischaemia-reperfusion injury in heart failure patients. Front. Physiol. 10, 1–9. 10.3389/fphys.2019.00264 30930798 PMC6428763

[B152] Toering T. J. Van Der Graaf A. M. Visser F. W. Buikema H. Navis G. Faas M. M. (2015). Gender differences in response to acute and chronic angiotensin II infusion: a translational approach. Phys. Rep. 3 (7), 1–11. 10.14814/phy2.12434 PMC4552520 26149279

[B153] Torres-Estay V. Carreño D. V. San Francisco I. F. Sotomayor P. Godoy A. S. Smith G. J. (2015). Androgen receptor in human endothelial cells. J. Endocrinol. 224 (3), R131–R137. 10.1530/JOE-14-0611 25563353 PMC4700832

[B154] Usselman C. W. Stachenfeld N. S. Bender J. R. (2016). The molecular actions of oestrogen in the regulation of vascular health. Exp. Physiol. 101 (3), 356–361. 10.1113/EP085148 26778523 PMC4947570

[B155] Vallabhajosyula S. Ponamgi S. P. Shrivastava S. Sundaragiri P. R. Miller V. M. (2020). Reporting of sex as a variable in cardiovascular studies using cultured cells: a systematic review. Faseb. J. 34 (7), 8778–8786. 10.1096/fj.202000122R 32946179 PMC7383819

[B156] Vicencio J. M. Yellon D. M. Sivaraman V. Das D. Boi-Doku C. Arjun S. (2015). Plasma exosomes protect the myocardium from ischemia-reperfusion injury. J. Am. Coll. Cardiol. 65 (15), 1525–1536. 10.1016/j.jacc.2015.02.026 25881934

[B157] Viswambharan H. Carvas J. M. Antic V. Marecic A. Jud C. Zaugg C. E. (2007). Mutation of the circadian clock gene Per2 alters vascular endothelial function. Circulation 115 (16), 2188–2195. 10.1161/circulationaha.106.653303 17404161

[B158] Vriens J. de Rooij L. P. M. H. Goveia J. Rohlenova K. Dumas S. J. Meta E. (2020). Single-cell transcriptome atlas of murine endothelial cells. Cell 180 (4), 764–e20. 10.1016/j.cell.2020.01.015 32059779

[B159] Wiesner C. Vliet V. Van. Butt E. Pavensta H. Sto M. Linder S. (2012). Lasp-1 regulates podosome function. PLoS One 7 (4), 1–10. 10.1371/Citation PMC3325968 22514729

[B160] Xiao J. Pan Y. Li X. H. Yang X. Y. Feng Y. L. Tan H. H. (2016). Cardiac progenitor cell-derived exosomes prevent cardiomyocytes apoptosis through exosomal miR-21 by targeting PDCD4. Cell Death Dis. 7 (6), e2277 10.1038/cddis.2016.181 27336721 PMC5143405

[B161] Xiao Q. Zhao X. Y. Jiang R. C. Chen X. H. Zhu X. Chen K. F. (2019). Increased expression of Sonic hedgehog restores diabetic endothelial progenitor cells and improves cardiac repair after acute myocardial infarction in diabetic mice. Int. J. Mol. Med. 44 (3), 1091–1105. 10.3892/ijmm.2019.4277 31524224 PMC6657988

[B162] Xue B. Beltz T. G. Guo F. Johnson A. K. (2018). Sex differences in maternal gestational hypertension-induced sensitization of angiotensin II hypertension in rat offspring: the protective effect of estrogen. Am. J. Physiol. Regul. Integr. Comp. Physiol. 314 (2), R274–R281. 10.1152/ajpregu.00216.2017 29046315 PMC5867674

[B163] Yin Y. Duan J. Guo C. Wei G. Wang Y. Guan Y. (2017). Danshensu accelerates angiogenesis after myocardial infarction in rats and promotes the functions of endothelial progenitor cells through SDF-1α/CXCR4 axis. Eur. J. Pharmacol. 814, 274–282. 10.1016/j.ejphar.2017.08.035 28864209

[B164] Youn S.-W. Li Y. Kim Y.-M. Sudhahar V. Abdelsaid K. Kim H. (2019). Modification of cardiac progenitor cell-derived exosomes by miR-322 provides protection against myocardial infarction through nox2-dependent angiogenesis. Antioxidants 8 (1), 18 10.3390/antiox8010018 PMC6356993 30634641

[B165] Yu M. Tsai S.-F. Kuo Y.-M. (2017). The therapeutic potential of anti-inflammatory exerkines in the treatment of atherosclerosis. Ijms 18 (6), 1260 10.3390/ijms18061260 PMC5486082 28608819

[B166] Zhang B. F. Jiang H. Chen J. Hu Q. Yang S. Liu X. P. (2019). Silica-coated magnetic nanoparticles labeled endothelial progenitor cells alleviate ischemic myocardial injury and improve long-term cardiac function with magnetic field guidance in rats with myocardial infarction. J. Cell. Physiol. 234 (10), 18544–18559. 10.1002/jcp.28492 30982985 PMC6617719

[B167] Zhang R. Lahens N. F. Ballance H. I. Hughes M. E. Hogenesch J. B. (2014). A circadian gene expression atlas in mammals: implications for biology and medicine. Proc. Natl. Acad. Sci. U.S.A. 111 (45), 16219–16224. 10.1073/pnas.1408886111 25349387 PMC4234565

